# Co-Regulation of Protein Coding Genes by Transcription Factor and Long Non-Coding RNA in SARS-CoV-2 Infected Cells: An In Silico Analysis

**DOI:** 10.3390/ncrna7040074

**Published:** 2021-11-29

**Authors:** Chinmay Saha, Sayantan Laha, Raghunath Chatterjee, Nitai P. Bhattacharyya

**Affiliations:** 1 Department of Genome Science, School of Interdisciplinary Studies, University of Kalyani, Nadia 741235, India; chinmaygensc19@klyuniv.ac.in; 2 Human Genetics Unit, Indian Statistical Institute, 203 B. T. Road, Kolkata 700108, India; laha.sayantansxcs2010@gmail.com (S.L.); rchatterjee@isical.ac.in (R.C.); 3 Department of Endocrinology and Metabolism, Institute of Post Graduate Medical Education & Research and Seth Sukhlal Karnani Memorial Hospital, Kolkata 700020, India

**Keywords:** COVID-19, lncRNA, PCG, transcription factors, pathways

## Abstract

Altered expression of protein coding gene (PCG) and long non-coding RNA (lncRNA) have been identified in SARS-CoV-2 infected cells and tissues from COVID-19 patients. The functional role and mechanism (s) of transcriptional regulation of deregulated genes in COVID-19 remain largely unknown. In the present communication, reanalyzing publicly available gene expression data, we observed that 66 lncRNA and 5491 PCG were deregulated in more than one experimental condition. Combining our earlier published results and using different publicly available resources, it was observed that 72 deregulated lncRNA interacted with 3228 genes/proteins. Many targets of deregulated lncRNA could also interact with SARS-CoV-2 coded proteins, modulated by IFN treatment and identified in CRISPR screening to modulate SARS-CoV-2 infection. The majority of the deregulated lncRNA and PCG were targets of at least one of the transcription factors (TFs), interferon responsive factors (IRFs), signal transducer, and activator of transcription (STATs), NFκB, MYC, and RELA/p65. Deregulated 1069 PCG was joint targets of lncRNA and TF. These joint targets are significantly enriched with pathways relevant for SARS-CoV-2 infection indicating that joint regulation of PCG could be one of the mechanisms for deregulation. Over all this manuscript showed possible involvement of lncRNA and mechanisms of deregulation of PCG in the pathogenesis of COVID-19.

## 1. Introduction

Coronavirus disease 19 (COVID-19) is caused by infection with severe acute respiratory syndrome corona virus 2 (SARS-CoV-2). A small fraction of infected individuals progress to severe conditions resulting in multi-organ failure which may be fatal [1]. Deregulated macrophages, innate and adaptive immunity result in hyperinflammatory responses, and enhanced expression of various cytokines, and chemokines have been observed in COVID-19 and reviewed [2]. The role of interferons (IFNs) as immune mediators in COVID-19 patients remains debated. Levels of IFN-I and IFN-III were dependent on anatomical sites, viral load, and severity. Increased level of IFN-III is observed in the upper airways of patients with a high viral burden but reduced disease risk or severity. In the lower airways of patients with severe COVID-19, IFNs are over-represented and show gene pathways associated with increased apoptosis and decreased proliferation [3].

### 1.1. Molecular Pathogenesis of COVID-19

SARS-CoV-2 enters the host cells by interaction of viral S protein with ACE2 receptor and cleavage of the S protein by host proteinase like TMPRSS2 [4]. The virus within the host cells uses stored energy and substances like nucleotides, amino acids, and lipids and hijacks the host’s replication and translation machinery resulting in viral replication and finally leading to cellular collapse. Macrophages in the immune system could phagocytose entire viral particles and hydrolyze the virus with various hydrolytic enzymes in the lysosomes [5]. If the virus escapes elimination by the host immune system and releases its RNA, the virus or the released RNA genome is recognized by various pattern recognition receptors and results in final activation of IFN and other proinflammatory cytokine genes by transcription factors IRFs and/or NFκB through various intermediate mediators. Secreted IFNs bind to receptors like IFNAR1 and IFNAR2, activate downstream JAK-STAT pathway to induce interferon-stimulated genes (ISGs) for their antiviral activity. IFN may also activate ISGs through MAPK-pathway, independent of JAK-STAT pathway. ISGs function as antiviral factors by inhibiting viral entry, viral replication, transcription, translation viral assembly, and release. Negative regulators, such as suppressors of cytokine signaling (SOCS), USP 18, and STAT3, may help IFN-induced cells to return to cellular homeostasis. Such cytokine responses induce the host’s immune system to clear the virus; however, if the immune system is overactivated, the cytokines might infiltrate the lung tissue and develop a cytokine storm leading to acute respiratory distress syndrome (ARDS) [6,7,8,9]. The escape of the antiviral response of the host is mediated through alteration of transcription of the host PCG as well as non-coding RNA genes; a detailed description of altered gene expression is discussed in the following sections [4,5]. Interactions of viral proteins with the host proteins might hijack host proteins and modify cellular pathways to evade host immune response and survival of viruses. Interaction of viral protein with host proteins might modulate functions of host proteins by (i) changing cellular localization, transport and interaction with other host proteins, (ii) changing activation of the host proteins by post-translational modifications, and (iii) degradation of the host proteins [7,10,11]. Within a short period of time since the COVID-19 pandemic started, several large-scale expression studies, mainly by RNA sequencing have been carried out to understand the pathophysiology of the disease and describe it.

### 1.2. Altered Expression of Genes

#### 1.2.1. Deregulated Protein Coding Genes in SARS-CoV-2 Infected Cells or Tissues from COVID-19 Patients and Their Biological Functions

Thousands of altered expressions of genes were identified from various tissues like lungs [12], nasopharyngeal swabs [13], PBMC [14], human induced pluripotent stem cell-derived cardiomyocytes (hiPSC-CMs) [15], human induced pluripotent hepatocytes or pancreatic cells or organoids [16] and cultured cells infected with SARS-CoV-2 [12]. Inappropriate inflammatory response defined by elevated cytokine, chemokine expression in the absence of Type I and III interferons has been observed in COVID-19 [12,17,18,19]. In monocytes from severely affected COVID-19, IFN-1 response, and TNF/IL-1β-driven inflammation co-existed and were absent in patients with milder COVID-19. It has been proposed that IFN-1 response plays a pivotal role in exacerbating inflammation in severe COVID-19 [14]. Increased expression of IFN-I responsive genes has been observed in SARS-CoV-2 infected intestinal organoids [20]. A similar result of robust IFN-I and pro-inflammatory response has also been observed in BALF of COVID-19 patients [21]. Several interferon responsive genes were upregulated in SARS-CoV-2 infected NBHE cells. This result indicates activation of antiviral IFN innate response in virus infected cells [22]. The apparent discrepancy of reduced IFN-1 response and higher expression of ISGs in PBMC was resolved by identifying transient low plasma IFN-α levels originated from lungs [19]. SARS-CoV-2 infection activates pro-inflammatory genes and interferon/cytokine signaling in these cells. Infection of SARS-CoV-2 in lung epithelium alters cell to cell communication patterns between lung epithelial cells and the immune system, and results in a dysregulated host immune response in COVID-19 [23]. SARS-CoV-2 infection induced a strong interferon-driven antiviral response and reduced transcription of ribosomal proteins. It has been demonstrated that host responses to SARS-CoV-2 are dependent on viral load and infection time course, with observed differences due to age and sex that may contribute to disease severity [13]. In severe COVID-19 patients, differentially expressed genes are enriched with adaptive cellular and humoral immune responses, cell cytotoxicity, chemotaxis, and apoptosis compared with those in moderate cases [24]. In summary, altered expression of genes might play important role in COVID-19 in IFN response, although remains debated. Deregulated genes are involved in diverse cellular functions like inflammation, immunity, apoptosis and others. Results described in these and additional studies show altered expression of IFN-responsive interferon stimulated gene (ISGs) and genes involved in inflammation and immunity.

#### 1.2.2. Long Non-Coding RNA

Long non-coding RNAs (lncRNA) are heterogeneous transcripts, longer than 200 nucleotides without the potential for coding proteins. Similar to protein coding genes (PCG), lncRNAs are also under transcriptional control of TF, chromatin modification, and promoter methylation. LncRNA interacts with chromatin, DNA, mRNA, protein, microRNA and modulates the levels of protein coding genes (PCG) at transcription, post-transcription, or post-translation levels. Interaction of lncRNA with DNA might result in DNA-RNA triple helices, facilitating or inhibiting the recruitment of transcription factors (TFs) and regulating expression of nearby genes (cis) or at distance (trans). Interactions of lncRNA with mRNA or protein may stabilize or destabilize the mRNA. Interaction of lncRNA with protein might alter the stability and localization of proteins and thus alter the expression of the genes when interacting with TF. Interaction with proteins involved in splicing might also indirectly level of mRNA. LncRNA has been implicated in diverse biological processes, functions, and pathways including transcription, mRNA splicing, apoptosis, immunity, inflammation by altering the level of proteins (reviewed in [25,26,27]).

##### Altered Expression of lncRNA in Cells Infected with Viruses Other Than SARS-CoV-2

Altered expression of lncRNA has been observed in viral infection and reviewed [28]. Infection with influenza virus (IAV), HIV, herpes simplex virus (HSV), hepatitis C virus (HCV), hepatitis B virus (HBV), severe acute respiratory syndrome coronavirus (SARS-CoV), and others alter the expression of host lncRNA and reviewed [29,30,31]. Altered expression of the lncRNAs in different viruses other than SARS-CoV-2 infected cells has been implicated in diverse cellular functions like antiviral activity by modulating viral replication and growth as well as expression of IFNs, and ISGs, possibly by interacting with transcription factors like STAT1, STAT3, NFκB, IRFs and has been reviewed recently [31]. Detail of the literature search for deregulation of lncRNA in viruses other than SARS-CoV-2 infected cells and their possible function is shown in Table S1.

##### Altered Expression of lncRNA in SARS-CoV-2 Infected Cells

Expressions of hundreds of lncRNAs including uncharacterized novel transcripts have been identified in SARS-CoV-2 infected cells [32,33,34,35], BALF from the COVID-19 patients [35,36], and PBMC from COVID-19 patients [36,37,38,39]). In most of the studies, re-analyses of expression data for lncRNA from published transcriptomic data, except in two studies [33,37] were carried out. Combining the all-published results, expression of 87 lncRNA was increased and 283 lncRNA was decreased only in one study. Increased expression of 21 lncRNA and decreased expression of 39 lncRNA were observed in more than one study. Expression of 26 lncRNA was altered in more than one experiment but in the opposite direction (Tables S2–S5). Validation of differentially expressed lncRNA by low throughput assays is absent in most of the studies. In some studies, differences in the expressions of lncRNAs in PBMC and BALF were observed. For example, MALAT1 was decreased in PBMC, while increased in BALF. Similarly, the expression of NEAT1 did not alter in PBMC but was increased in BALF [36]. This difference in PBMC and virus infected cells could be due to the indirect effects of virus infection in PBMC and infected cells. Differential expression of lncRNA in PBMC between severe and mildly affected COVID-19 patients has also been reported. It has been observed that increased level of LINC02207, LINC01127 was associated with the severe COVID-19 group, whereas LINC02084, LINC02446, LINC00861, LINC01871, and ANKRD44-AS1 were associated with the mild COVID-19 group [38]. PVT1 was downregulated in severe COVID-19 patients compared with non-severity [37]. Given thousands of lncRNA coded by the human genome and the lack of consensus of the deregulated lncRNA in SARS-CoV-2 infection, further studies are necessary. Additionally, the mechanism (s) of deregulation of lncRNA, especially by TFs is poorly known.

##### Possible Functional Role of the Deregulated lncRNA in Viral Infection

The functional role of the deregulated lncRNA in the pathogenesis of the disease and mechanism (s) of deregulation are poorly known. Analyzing PCG co-expressed with the lncRNA or identifying interacting partners of the lncRNA from various databases and enrichment analyses inferred the possible role of the deregulated lncRNA in the pathogenesis of COVID-19. Such studies revealed that increased expression of LASI (antisense strand to ICAM-1, not annotated in NCBI and Ensembl database), TOSL (TNFAIP3-opposite strand lncRNA, not annotated in NCBI and Ensembl database) and NEAT1 might be involved in immunomodulation [33]. Analyzing the PCGs that modulate the expression of genes by acting in cis or trans as evident from the correlation of lncRNA-PCG, it has been inferred that MALAT1 and NEAT1 possibly contributed to the inflammation developed in the SARS-CoV-2 infected cells [36]. The role of deregulated lncRNA in total lymphocytes, T cell, CD4 + T cell and B cell counts, increase procalcitonin and CRP have been inferred from the clustering of differentially expressed lncRNA and their associated PCGs. Decreased lncRNA thus has potential immunological functions [37]. Many pathways related to viral infection, inflammation, and immune functions were enriched with deregulated lncRNA interacting partners in SARS-CoV-2 infected cells. Possible regulation of the deregulated lncRNA by interferon regulatory factors (IRFs) and STATs and has been described. It has been shown that any one of the transcription factors IRF1, IRF4, STAT1, STAT3, and STAT5A had experimentally determined binding regions within the putative promoters (−5 kb to +1 kb of the transcription start sites) of deregulated lncRNAs in at least two independent cell lines/conditions. Besides, it has been shown that more than 400 miRNAs could interact with the deregulated lncRNA as evident from the NPInter v4.0 database [34]. Expressions of different cytokines were altered in COVID-19 patients. Possible interaction of these cytokines related to cytokine storm with lncRNA was obtained from LncRNA2 Target [40] and observed that several lncRNA might interact with these cytokines; expression of these lncRNA was not known from this study [41].

Diverse biological processes and pathways associated with deregulated lncRNA in SARS-CoV-2 infection have been reported and reviewed. These pathways include oxidative stress, immune responses [42], NLRP3 inflammasome [43,44], TNF signaling pathway, IL-17 signaling pathway, cytokine-cytokine receptor interaction, NOD-like receptor signaling pathway, Influenza A, chemokine signaling pathway, RIG-I-like receptor signaling pathway, MAPK signaling pathway, NF-kappa B signaling pathway, Toll-like receptor signaling pathway, AGE-RAGE signaling pathway in diabetic complications, FoxO signaling pathway, apoptosis, epithelial cell signaling in Helicobacter pylori infection and cellular senescence [35], viral defense response, innate immune response, and inflammatory response [9].

##### Mechanism of Action of lncRNA in Viral Infection

LncRNA has antiviral activity, modulates viral replication and growth, and alters IFN and/or ISG expression possibly by interacting with transcription factors STAT1, STAT3, NFκB, and IRFs in response to several viral infections and has been reviewed recently [8,45,46]. For example, increased expression of NEAT1 in response to Hantaan virus infection enhances robust IFN responses by binding to the promoter of IFNB and increasing the expression [47]. IFN regulated lncRNA is shown in the Table S1 (column E). Increased expression of NEAT1, observed in many virus infected cells, has been shown to modulate the expression of IL8 by relocating SFPQ from its promoter and recruiting SFPQ into the paraspeckles [48]. Antiviral activity of NEAT1 could be mediated through formation of paraspeckles. Paraspeckles could sequester paraspeckle-localizing proteins and RNA and modulate their functions outside the paraspeckles, thus acting as molecular sponges [49,50]. Paraspeckles are induced by IAV, HSV [48], and HIV [51].

NEAT1 might promote IFN responses by acting as positive feedback for Retinoic acid-inducible gene I (RIG-I) encoded by the DDX58, signaling. Increased NEAT1 by interacting with SFPQ, relocates SFPQ from the promoters of RIG-I and DDX60 to paraspeckles. Thus, NEAT1 removes the transcriptional inhibitory effects of SFPQ on RIG-I and DDX60, resulting in increased expression of transcriptional factor IRF7 which in turn induced the expression of IFN and NEAT1 [47]. NEAT1 may also activate IRF3 through the formation of multi-subunit complexes with HEXIM1. NEAT1-HEXIM1 complex interacts with cGAS sensor and its partner PQBP1 and releases proteins from paraspeckle. Released proteins are recruited to STING and activate IRF3 producing type 1 IFN. These results indicate that NEAT1 has a critical role in the antiviral response of IFN through (i) RIG-I signaling and (ii) cGAS-STING-IRF3 pathway as reviewed [8].

Many deregulated lncRNA, observed in different viral infections, has been shown to modulate viral growth by regulating ISGs possibly due to interactions of lncRNA with host proteins. Contributions of lncRNA in the alteration of host protein coding genes and diverse biological processes and pathways as observed in different other conditions [25,27] are not known fully. LncRNA also regulates innate immunity in response to viral infection. This has been described in detail in Supplementary Sections S1.1.1–S1.1.3.

##### Deregulation of microRNA and Circular RNAs in SARS-CoV-2 Infected Cells

Altered expression of microRNA, the negative regulator of PCG, has been observed in different viral infections including SARS-CoV-2. These deregulated host miRNA by targeting 3′-untranslated regions of host PCG and could modify innate immunity, inflammation, viral replication, etc., and were reviewed recently [52,53,54]. Various host microRNA has been identified by computational methods to bind with the SARS-CoV-2 RNA Genome [55,56,57,58]. SARS-CoV-2 might code microRNA that could regulate the host gene [59,60,61] These in silico analyses and limited experimental results revealed the role of microRNA as a potential marker for SARS-CoV-2 infection, as well as a target for the treatment of COVID-19 [62]. Besides, Circular RNA (circRNA), covalently closed long noncoding RNA without 3′-poly(A) tail and 5′ cap, encoded by the host genomes and different viruses including SARS-CoV-2 have been identified computationally. The circRNAs have diverse functions; they can function as microRNA sponges, encode proteins or interact with other proteins [63,64,65]. Differential expression of hundreds of circRNAs has been identified in the PBMC of COVID-19 patients [66]. Interaction of microRNA with lncRNA through sequence complementation sequesters microRNA and prevents its interaction with mRNA [67]. In silico competing endogenous RNA (ceRNA) analysis revealed that in humans, the decrease of microRNA activity caused by viral RNAs can lead to a perturbation of vesicle trafficking and the inflammatory response [68].

##### Interaction of lncRNA and microRNA with SARS-CoV-2 Genome

Using the computational prediction tool LncTar [69] interactions of differentially expressed lncRNA with SARS-CoV-2 genome have also been predicted. Differentially expressed PVT1 and HOTAIRM1 showed high affinity for binding with the ORF1ab gene that encodes the NSP1, NSP2 and NSP3 [36]. The strong interaction of H19 with the 5′-UTR of the viral genome as well as the gene coding for spike protein was predicted. MIAT and APOA1-AS are also predicted to interact with the viral genome although with lesser affinity than that of H19 [70]. Interactions of several host miRNA with the 5′ leader sequence and 3′-UTR of the viral genome, as well as the viral gene coding for spike protein have been predicted [70] Similar results of targeting viral genome by the host microRNA has also been published [67]. Experimental validation of this prediction and the consequence of such interactions remains to be found out.

### 1.3. Transcription Factors in SARS-CoV-2 Infection

Various TF especially IRFs (IRF1-IRF9) and Transducers and Activators of Transcription (STAT1-STAT6) play important roles in viral infection including SARS-CoV-2 infection through the regulation of IFN production and potentiate the expression of antiviral genes including inflammatory cytokine genes [71,72,73]. IRFs are also involved in diverse cellular functions including innate immunity, cell cycle progression, apoptosis, and tumor suppression [74]. Activation of STAT proteins downstream to many cytokine receptor families triggered by elevated cytokines has been observed in SARS-CoV-2 infected cells and reviewed. Hospitalized COVID-19 patients showed increased levels of various cytokines that in turn activate multiple cytokine receptor families and downstream Janus Kinases (JAKs) and STAT proteins to eradicate the SARS-CoV-2 pathogen and restore immune homeostasis. Cytokines utilizing IFN-I, IFN-II, and G-Protein Coupled Receptors propagate direct signals through JAKs and STATs and many have been linked to COVID-19 disease severity [71]. ISGF3, a tripartite transcription factor formed by high levels of IRF9, STAT1, and STAT2, induces the expression of hundreds of ISGs. In silico analysis revealed that several host TFs induced by phosphorylated ISGF3 and unphosphorylated ISGF3 (U- ISGF3) could bind near the transcription start sites of viral genes coded by the SARS-CoV-2. For example, TFs induced by U- ISGF3 like SHOX, JUN interact with SARS-CoV-2 genome coding for N, phosphorylated ISGF3 induced Stat5a binds to the genome coding for the spike protein S. It has further been observed that binding sites of 22 TF like E2F1, E2F4, E2F6, EGR1, KLF5, SP8, TBXT were present only in the sequence of SARS-CoV-2 and were absent in coronavirus RaTG13. These TFs are involved in IFN, retinoic acid signaling, regulation of transcription by RNA polymerase II and are likely to involve in viral replication. Once experimental evidence is provided, the mechanism of interaction of TFs with the viral genome would provide an alternative pathway for the disruption of IFN signaling in host cells, as well as retinoic acid depletion syndrome leading to cytokine storms in COVID-19 [75]. Transcription factor NF-κB comprises of proteins p65 (RelA), RelB, c-Rel, p105/p50 (NFκB1), and p100/52 (NFκB2). NFκB in the inactive form resides in the cytosol and interacts with the IκB proteins. The phosphorylation of IκBs by IKK leads to nuclear translocation of NFκB and binds to their target promoters activating transcription of different genes involved with inflammation, cell proliferation, and apoptosis [76]. Hyper activation of the NFκB pathway involves the survival, activation, and differentiation of innate immune cells, inflammatory T cells and has been implicated in the pathogenesis of severe/critical COVID-19 patients [77]. Other TFs like NFE2L2/NRF2, known to regulate the expression of antioxidant proteins [78], KLF2, a master regulator of vascular homeostasis [79], HIF1A, known to regulate genes in response to hypoxia [80], MYC [81], RUNX1 [82], FOXC1, GATA2, YY1, E2F1, NFIC, FOXL1 and SRF [83,84] have been implicated in COVID-19.

#### Regulatory Network: TFs-LncRNA-PCG

Complex molecular interactions of TF, lncRNA, microRNA, and other epigenetic changes control gene expression. In the simplest form, TFs bind to the regulatory sequences (promoters and enhancers) and activate or repress the expression of PCGs, microRNA, or lncRNA. A regulatory network is composed of a complex web of molecular factors that interact with each other and with genes in order to control gene expression. In the simplest form of gene-regulatory network, TFs bind to regulatory sequences and their interactions result in the induction or repression of the gene expression [85]. Introduction of post-transcriptional regulation of PCG by microRNA (Figure S1), a negative regulator of PCG results in the complex regulation of PCG and studied in detail by several investigators [86,87,88,89]. In such a mixed network of TF-microRNA-targets, several recurrent regulatory patterns can be detected, called network motifs, similar to that obtained with the TF-target network [90]. Among these motifs, microRNA-mediated feedforward loop (FFL), where TF regulates a miRNA and, together with it, a set of common target genes of TF and microRNA. Over-presentation of FFL in many regulatory networks supports the notion that it might play an important regulatory role. Depending on the ability to activate or repress the expression of the target PCG or microRNA, the FFL could be classified as coherent and incoherent. In the coherent FFLs, TFs could activate or repress both microRNA and the target PCG, while in the incoherent FFLs, expression of microRNA and PCG are in opposite directions (Figure S1) [91]. MicroRNA being a negative regulator of PCG, in coherent FFLs, the expression of microRNA and target PCG are highly anticorrelated (in opposite direction), while in incoherent FFLs expression of them are highly correlated (in the same direction). For example, in a coherent FFL TF induces the miRNA expression; the same TF represses the joint target PCG. This condition enforces mutually exclusive expression as the ones observed in several cases. Thus, microRNA can help the transcriptional repression of a target protein that should not be expressed in a particular cell type or condition acting as a post-transcriptional “failsafe” control. Incoherent FFL can promote high PCG expression in microRNA expressing cells, indicating that microRNA might have a “fine-tuning” function, maintaining the protein level in the appropriate functional range [92].

Regulatory networks or motifs of TF-lncRNA-Targets are likely to be more complex, as lncRNA can directly interact with DNA/chromatin and represses or activates transcription, and interaction with mRNA and protein might stabilize or destabilize mRNA and protein. Interaction of lncRNA with microRNA might sequester/destabilize the microRNA and affect the targets of the microRNA (Figure S1). Possible FFLs in the TF-lncRNA-joint targets network is shown in the Figure S2. FFL consisting of TFs, lncRNA, and targets has not been widely studied. FFL consisting of RNA binding protein HuR, lncRNA HOTAIR and microRNA has been identified [93]. BCYRN1 has been shown to form FFL with an hnRNPA1/WNT5A/VEGFR3 for the promotion of lymphatic metastasis of breast cancer [94]. Feed-forward loops consisting of TF-miRNA-lncRNA in cancers have been identified [95,96].

### 1.4. Knowledge Gap

Altered expression of PCG and lncRNA has been observed in SARS-CoV-2 infected cells as well as in tissue samples from COVID-19 patients in many studies. Given thousands of lncRNA are coded by the human genome, deregulation expression of hundreds of lncRNA only has been identified and varies from one study to another. Functional consequences of the deregulated lncRNA are not known fully. Even though deregulation of several TFs is observed, their target lncRNA and PCG are not known. LncRNA is known to modulate the expression of genes/proteins at the transcription and post transcription level; co-regulation of the altered PCG by TRF and lncRNA in viral infection remains unknown.

## 2. Material and Methods

**Figure 1:**
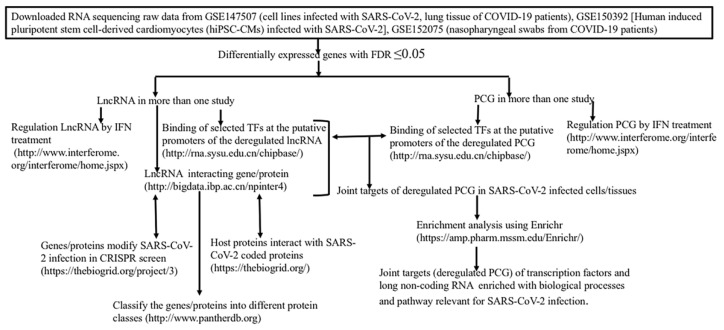
The overall flow diagram is followed in the manuscript. To find out the transcription factors (TFs), especially IFR1-IFR5, IRF8, IRF9, STAT1-STAT4, STA5A, STAT6, NFKB1, NFKB2 and RELA/p65 (for justification for the choice please see Section 2.3) that might regulate the expression of the genes, we searched ChIPBase database. Similarly, the Interferome database was used to find out whether genes could be regulated by IFNs. Interacting partners of deregulated lncRNA obtained from NPInter v4.0 were further used for identifying the functional role of the genes/proteins in SARS-CoV-2 infection from BioGRID and PANTHER. To identify the common targets of TFs and deregulated lncRNA, we compared (shown as a double headed arrow) lncRNA interacting gene/protein and deregulated PCGs that were targets of common TFs. Finally the enrichment of the common targets of TFs and lncRNA using Enrichr for functional annotation of the co-regulated targets. IP addresses used are included. For details please see the text.

The overall approach to identify the joint targets of lncRNA and TFs deregulated in SARS-CoV-2 infected cells by using diverse publicly available resources is shown in Figure 1.

### 2.1. Data from Gene Expression Omnibus (GEO)

Deregulation of protein coding genes has been observed in many studies. In SARS-CoV-2 infected cell lines and lungs of COVID patients altered expression of genes has been reported ([12]; GSE147507)). Altered expression of genes in human pluripotent stem cell-derived cardiomyocytes (hiPSC-CMs) infected with SARS-CoV-2 ([15,97] GSE150392), in nasopharyngeal swabs of COVID-19 ([13]; GSE152075), in hiPSC-pancreatic cells/organoid and hiPSC-hepatocytes/organoid infected with SARS-CoV-2 [16] and PBMC of COVID-19 patients has been reported. The raw RNA sequencing data were downloaded from Gene Expression Omnibus (GSE147507, GSE150392, and GSE152075). Detail of different cells infected with SARS-CoV-2 in GSE147507 has been described earlier [34]. In GSE150392, human induced pluripotent stem cell-derived cardiomyocytes (hiPSC-CMs) infected with SARS-CoV-2, a model system to examine the mechanisms of cardiomyocyte-specific infection by SARS-CoV-2, was used. RNA sequencing in nasopharyngeal swabs from 430 individuals with SARS-CoV-2 and 54 negative controls was carried out [13]. Summary of the different data used to determine the differential expression of genes is shown in the Table S54.

Methodologies used to determine the differential expression of lncRNA and PCG using Bioconductor package edgeR were similar as described in detail earlier. Low abundant genes were filtered out from the dataset [34]. False discovery rate FDR) was determined by Benjamini–Hochberg method. FDR ≤ 0.05 was considered to be significant. Other important parameters like the magnitude of the fold changes with respect to the controls are given in the column logFC, and the average counts per million of the genes in the samples, as provided in the log(cpm) column in the output files. In addition, we downloaded the Supplementary Tables from 2 published papers [14,16]. We annotated the genes using Ensembl Biomart (http://www.ensembl.org/biomart/martview/, Ensembl gene 104, Human genes, GRCh38.p13; accessed on 1 July 2021).

### 2.2. Interacting Partners of Long Non-Coding RNA

We downloaded experimentally determined and curated physical interaction data of lncRNA with DNA, mRNA, microRNA, proteins, and others from NPInter v4.0 (http://bigdata.ibp.ac.cn/npinter4, accessed on 1 July 2021) [98]. The database catalogs experimentally derived interactions, collected manually from publications in peer-reviewed journals, and annotated using other databases like NONCODE, miRBase, and UniProt. Annotation of genes in NPInter database was further annotated using NCBI database (ftp://ftp.ncbi.nih.gov/gene/DATA/GENE_INFO/Mammalia/, accessed on 1 July 2021).

#### 2.2.1. Protein Class of lncRNA Interacting Proteins

To gather general information of the genes/proteins that interact with deregulated lncRNA or deregulated genes we utilized the protein classification systems in PANTHER (http://www.pantherdb.org, accessed on 1 July 2021) and described [99]. Out of 20,595 PCG annotated in the database, 12,043 genes were broadly classified into 23 protein classes. Broad protein classes include extracellular matrix protein (PC00102), cell adhesion molecule (PC00069), nucleic acid metabolism protein (PC00171), gene-specific transcriptional regulator (PC00264), defense/immunity protein (PC00090), translational protein (PC00263), chromatin/chromatin-binding, or -regulatory protein (PC00077) and others.

#### 2.2.2. SARS-CoV-2 Coded Protein Interacting Partners of Host Proteins

Viral proteins coded by SARS-COV-2 are known to interact with a large number of host proteins as detected by large-scale, as well as low throughput assays and cataloged in BioGRID (BioGRID https://downloads.thebiogrid.org/File/BioGRID/Latest-Release/BIOGRID-PROJECT-covid19_coronavirus_project-LATEST.zip, accessed on 24 June 2021). We downloaded this data and curetted for SARS-CoV-2 coded proteins only in humans and mice.

#### 2.2.3. Host Proteins Identified for Modulation of SARS-CoV-2 by CRISPR Screens

To identify the host genes that might modulate the growth and survival of SARS-CoV-2, genome-wide CRISPR loss-of-function screens were carried out by several investigators [100,101,102,103,104,105,106,107] and observed many host genes modulate the infection. The host genes that modulate the infection are cataloged in BioGRID (https://thebiogrid.org/project/3, accessed on 1 July 2021). This data was obtained from (https://orcs.thebiogrid.org/Search?searchType=key&search=coronavirus|sars|mers&organism=all&pp=3, accessed on 1 July 2021).

#### 2.2.4. Functional/Genetic Interaction of Long Non-Coding RNA: LncRNA2Target DATABASE v3.0 

Interaction of lncRNA may influence the transcription of the target genes when interacting with DNA/chromatin. If lncRNA interacts with mRNA, it may alter the stability of mRNA and/or transport of the mRNA at a post-transcriptional level. Interaction with the protein can modulate the stability, trafficking of the protein, splicing, etc. Functional interaction of lncRNA is not known for most lncRNA. In the LncRNA2Target database (http://bio-annotation.cn/lncrna2target/index.jsp, accessed on 1 July 2021), interaction of lncRNA with gene/protein including functional interactions are catalogued. In several cases, knockdown of the lncRNA followed by high throughput or low throughput expression of target genes were provided. This data provides functional/genetic interactions of lncRNA with protein coding genes. This database provides a web interface through which the targets of a particular lncRNA or for the lncRNAs that target a particular gene could be obtained. Data in the database can be downloaded in bulk or searched for a query lncRNA. In the current version of the database (LncRNA2target v3.0) about 33,000 gene targets including microRNA and PCG are cataloged for 120 human lncRNA. One hundred and fifty mouse lncRNA could modify the expression of 22,407 genes [40].

#### 2.2.5. Interferon Regulated Genes

To find whether the deregulated lncRNA or PCG was responsive to different IFN treatments, we utilized the Interferome (http://www.interferome.org/interferome/home.jspx, accessed on 1 July 2021) database. This database contains IFN (type I, II, and III) regulated genes, defined as significantly up or downregulated relative to control untreated samples, manually curetted from publicly available microarray datasets. A list of genes in the input with specific parameters like type and concentration of IFN, time of exposure, specific tissue, and cut of values of fold change provides a list of genes regulated by INF [108]. We used all three types of IFN in all tissues and ±1.5 folds change as the criteria for interferon regulated genes only in human cells.

### 2.3. Binding of Transcription Factors at the Putative Promoters of PCGs and lncRNAs

To identify the binding of IFR1-IFR5, IRF8, IRF9, STAT1-STAT4, STA5A, and STAT6, MYC, NFKB1, NFKB2 and RELA/p65 at the putative promoters of deregulated PCG and lncRNA, we used ChIPBase database at http://rna.sysu.edu.cn/chipbase/ (accessed on 1 July 2021) as described earlier [34]. IRFs and STATs are known to induce expression of antiviral genes [109,110]; MYC interacts with MALAT1, and NEAT1 [111], and activation of MYC has been observed in IAV infection [112] as well as proposed to target deregulated genes in SARS-CoV-2 infected cells [81]. NFKB1, NFKB2 and RELA/p65, implicated in viral replication as well as antiviral innate immune responses [113,114]. ChIPBase v2.0 is an open database that catalogs experimentally binding of TFs transcription factors determined by chromatin immune-precipitation followed by sequencing (ChIP-seq) and curetted at different regions of PCG and lncRNA [115]. Experimentally determining binding of the TFs at the putative promoters [−5 Kb to +1 Kb of transcription start sites (TSS) of the genes] of PCG and lncRNA was downloaded from ChIPBase database at http://rna.sysu.edu.cn/chipbase/, accessed on 1 July 2021. Different cell lines and conditions used have been described earlier [34].

### 2.4. Association of Genes/Proteins with Biological Processes Defined by Gene Ontology and KEGG Pathways

To identify possible functional implications of the coregulated PCG by TFs and lncRNA, we used the online facility Enrichr at https://amp.pharm.mssm.edu/Enrichr/ (accessed on 1 July 2021) [116] as described earlier [34].

### 2.5. Cytoscape Representation of Interaction

For visualization of molecular interaction networks, we have used an open-source software platform-Cytoscape (version 3.9.0) [117].

### 2.6. Statistical Analysis

For analysis of raw sequencing data from GSE147507, GSE150392 and GSE152075 we used the Bioconductor package edgeR. Genes that had a false discovery rate (FDR) ≤ 0.05 were considered to be significant. For enrichment of genes/protein with different biological processes and pathways, we used the online facility Enrichr at https://amp.pharm.mssm.edu/Enrichr/, accessed on 1 July 2021. Given a set of genes as input, this database returns results of enriched pathways with significant levels (*p*-values and adjusted *p*-values with multiple testing corrections). All statistical analyses were performed using Graph pad prism software (Version 8, San Diego, CA, USA).

### 2.7. For Comparison of Different Set of Genes/Proteins

We Used Online Facility at http://bioinformatics.psb.ugent.be/webtools/Venn/, accessed on 1 July 2021.

## 3. Result

### 3.1. Deregulated Long Non-Coding RNA in Different Tissues from the COVID-19 Patients and SARS-CoV-2 Infected Cells

In our present analysis, we identified decreased expression of 33 lncRNA as well as 33 lncRNA in more than one study. The summary of the result is shown in Table 1. Elaborate data is shown in (Tables S6–S9). We also observed 232 deregulated lncRNA in the present study in only one experiment (Table S8). Increased expression of EGOT, EPB41L4A-AS1, LINC00605, MALAT1, MIAT, NEAT1 and decreased expression of TP53TG1 has been reported by us earlier [34]. Combining our earlier result, increased expression of 47 lncRNA was increased and 36 lncRNA was decreased in more than one experiment. Taken together, deregulation of 83 lncRNA was identified by re-analyzing the data published in large scale RNA sequencing analysis in cell lines and organoids infected with SARS-CoV-2, as well as in tissues from COVID-19 patients in more than one experimental condition (Table 1). Among 66 deregulated lncRNA observed in the present study, expression of 17 lncRNA in the same direction has been reported by others; expression of 39 lncRNA has not yet been published and expression 10 lncRNA has been shown to alter in opposite direction (Table S9). Very recently it has been shown that that NEAT1 and MALAT1 are highly expressed in saliva and nasopharyngeal swab samples of COVID-19 patients [118] confirming the result in the present study.

#### 3.1.1. Interacting Partners of Deregulated lncRNA

**Figure 2:**
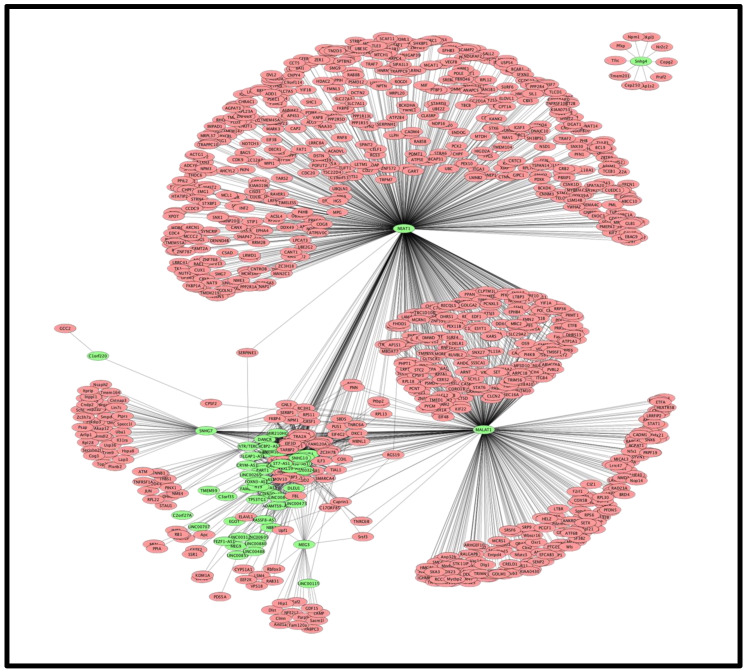
Cytoscape representation of the lncRNA-target interactions. Nodes in green color filled circles represent the lncRNA while the nodes in magenta color filled circles represent the target proteins/genes. Edges (lines joining the nodes) represent the interaction of lncRNA with genes/proteins obtained from NPInter v4.0.

LncRNA is known to interact with DNA/chromatin, mRNA, protein, and microRNA. To find out the functional implication of the deregulated lncRNA, we collected data for the interacting partners of the deregulated lncRNA from NPInter v4.0 [98]. It was observed that 44 lncRNA whose expression was increased could target 3178 protein coding genes at DNA, mRNA, and protein levels; interaction with protein was the most common mode of interactions. MLAT1 and NEAT1 had the highest number of interacting partners. It was further observed that 28 downregulated lncRNA had 246 targets; the same gene/protein could be the target of upregulated and down regulated lncRNA. All together 3228 unique genes/proteins are targets of the deregulated 72 lncRNA; no target was observed for decreased ARIH2OS, DGCR5, LINC00526, LINC00893, MIR1915HG, SLC22A18AS, SLC25A21-AS1, SNHG32 and increased HCG11, MIR497HG, and ZSWIM8-AS1 in the NPInter v4.0 database. NEAT1 interacts with a maximum number of genes/proteins (2460) followed by MALAT1 (1365). These two lncRNA together target about 93% (2951/3178) of all targets of deregulated lncRNA (Tables S10–S13). Cytoscape representation of lncRNA-target interaction is shown in Figure 2.

#### 3.1.2. Protein Classes of the lncRNA Interacting Partners

**Figure 3:**
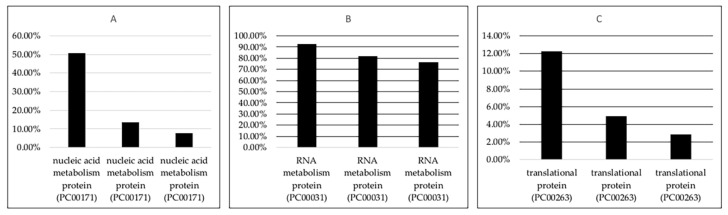
Bar diagram showing the protein classes nucleic acid metabolism (PC00171) (**A**), RNA metabolism (**B**) and translational proteins (**C**) of the targets of decreased lncRNA, increased lncRNA and the proteins coded by the human genome (from left to right). Representative protein classes are shown in the bottom. Increased proportion of the proteins among the targets of lncRNA was statistically significant (*p* < 0.05).

Protein classification of the lncRNA interacting proteins using PANTHER (http://pantherdb.org/geneListAnalysis.do, accessed on 1 July 2021) revealed that targets of deregulated lncRNA are associated with protein classes Nucleic acid metabolism protein (PC00171), RNA metabolism protein (PC00031), gene-specific transcriptional regulator (PC00264), translational protein (PC00263) and many others. The representative result is shown in Figure 3 and Table S55.

This result shows that different transcription factors, splicing factors, proteins involved in translation were associated with deregulated lncRNA (Table S56) and the detailed result is shown in Tables S14 and S15. This analysis shows that deregulated lncRNAs might alter splicing, gene expression and protein synthesis.

#### 3.1.3. Interacting Partners of lncRNA Are also Interacting Partners of Viral Proteins Coded by SARS-CoV-2

**Figure 4:**
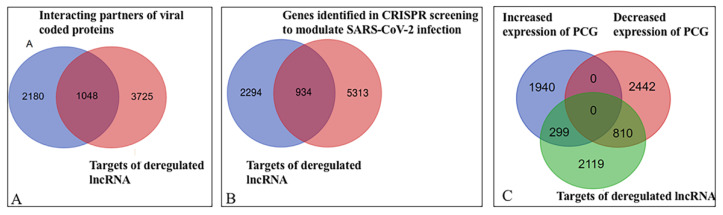
Common proteins among host interacting partners of SARS-CoV-2 coded proteins and targets of deregulated lncRNA (**A**), (**B**) represents the common proteins among genes/proteins identified to modulate SARS-CoV-2 infection and targets of deregulated lncRNA. (**C**) represents common PCGs altered in SARS-CoV-2 infected cells or in tissues from COVID-19 and targets of deregulated lncRNA.

**Figure 5:**
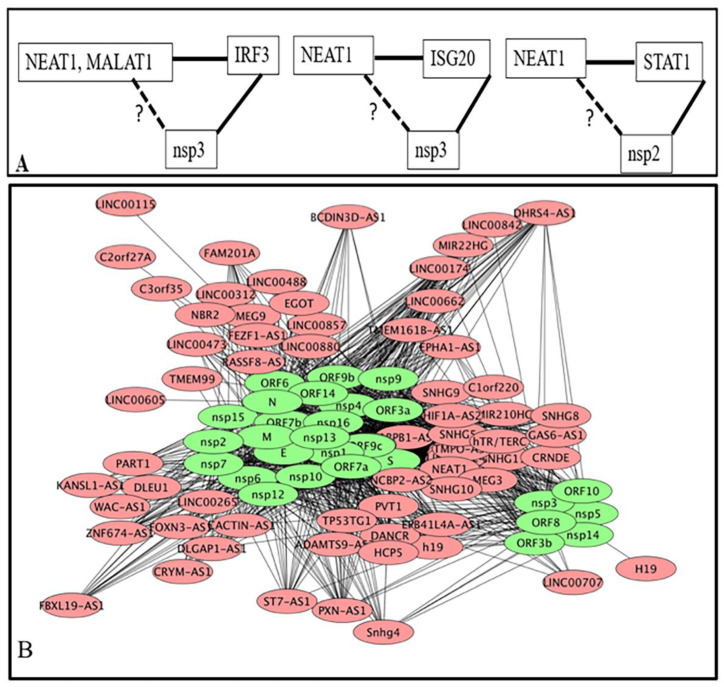
(**A**): Specific examples of indirect interaction of lncRNA (shown by the broken lines) with the viral proteins by localizing/interacting with common host proteins. (**B**): Cytoscape representation of lncRNA-viral protein interactions based on the interaction of lncRNA-genes/proteins from Figure 2 and proteins coded by the virus and lncRNA interacting proteins (Figure S3).

Viral proteins interact with host proteins to manipulate the host defense system, as well as the growth and replication of the virus. Interaction of lncRNA with the DNA/chromatin, mRNA, miRNA, and protein is known [98]. In the absence of information on interactions of lncRNA directly with a viral protein, we attempted to find out whether lncRNA interacting partners were also targets of viral proteins. About 5000 host proteins are the target of 30 SARS-CoV-2 coded proteins (Table S16). For example, viral E and M protein interact with 867 and 1615 host proteins respectively. Among the lncRNA interacting protein/gene (3228), 1048 genes/proteins were common with viral interacting proteins (Figure 4A), a viral protein targets many host proteins and a host protein may be the target of many viral proteins (Tables S17–S19). These common proteins interact with the SARS-CoV-2 coded proteins. Cytoscape representation of the interactions of lncRNA interacting proteins with the vital proteins is shown in the Figure S3. We combined the interactions of lncRNA with host proteins (Figure 2) and Figure S3 to obtain possible interactions of lncRNA with viral proteins. For example, IRF3 was a target of MALAT1 and NEAT1; non-structural protein nsp3 of SARS-CoV-2 could also target IRF3 [119]. This interaction was conserved in other corona viruses also [120]. NEAT1 interacts with interferon-stimulated gene ISG20 which is also a target of ORF9b (BioGRID). STAT1 is a target of MALAT1 that is also a target of nsp2. STAT6 is a common interacting partner of MALAT1 and NEAT1 as well as viral coded protein nsp9 (Figure 5A). Thus, MALAT1 and NEAT1 may interact/localize with IRF3, NEAT1 may interact with nsp3 and nsp3. Cytoscape representation of lncRNA with SARS-CoV-2 coded protein is shown in Figure 5B and summarized in Figure S3. This result shows that lncRNA could co-localize/interact with host proteins associated with the viral coded proteins and might modulate the function of host proteins. It remains to be found out how lncRNA contributes to the interaction of viral proteins with host proteins and modulates the functions of the host after infection of SARS-CoV-2.

#### 3.1.4. Host Genes Involve in SARS-CoV-2 Infection as Determine by Genome-Wide CRISPR Loss-of-Function Screens and Their Interactions with Deregulated lncRNA

**Figure 6:**
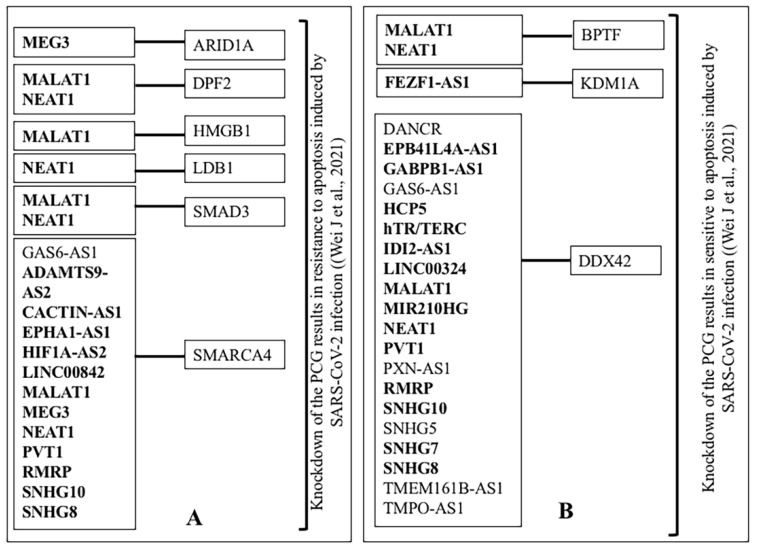
Representative result of CRISPR screening data from [106] is shown. Effects of knockdown of interacting partners of deregulated lncRNA in SARS-CoV-2 infected cells. Horizontal lines connecting two boxes indicate the interaction. LncRNA in bold face represents the increased expression in SARS-CoV-2 infected cells, (**A**): resistance to apoptosis, (**B**): sensitive to apoptosis. The same lncRNA interacting with different PCG might act as pro-viral or antiviral.

Genome-wide CRISPR loss-of-function screens identified 6248 genes to modulate SARS-CoV-2 infection [100,101,102,103,104,105,106,107]. We downloaded the data from BioGRID and compared it with the lncRNA interacting partners. Altogether out of 3228 targets of deregulated lncRNA, the knockdown of 934 targets modulate the infection of SARS-CoV-2 (Figure 4B). The detailed result is shown in Tables S20 and S21, B. For example, the knockdown of host PCG, MED16, NDOR1, SMG5 that interact with both MALAT1 and NEAT1 made cells sensitive to apoptosis induced by SARS-CoV-2 infection [106]. It has been observed that the knockdown of MYC, RELA, and STAT1 modulates the outcome of SARS-CoV-2 infection by modulation of apoptosis ([106], BioGRID). MYC was an interacting partner of deregulated lncRNA H19, hTR/TERC, MALAT1, NEAT1, and PVT1; RELA was a target of MALAT1 and NEAT1. Similarly, STAT1 interacted with MALAT1. The representative result of knockdown of lncRNA interacting proteins/genes and modulation of apoptosis induced by SARS-CoV-2 is shown in Figure 6.

#### 3.1.5. Interferon Responsive lncRNA

**Figure 7:**
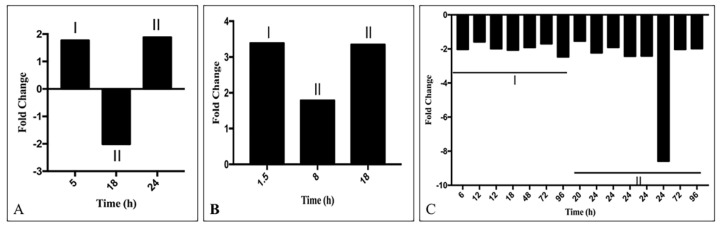
Representative result of changes in the expression of lncRNA NEAT1 (**A**), MALAT1 (**B**) and DANCR (**C**) at different time points by treatment with IFN-I (denoted by I) and IFN-II (denoted by II) as obtained from Interferome database (http://www.interferome.org/interferome/home.jspx, accessed on 1 July 2021).

Abnormal IFN response is an important pathological condition in COVID-19 (for a recent reference [3]). Deregulated IFN might play a role in the deregulation of gene expression. Treatment with IFNs modulates the expression of many PCG in vitro [121], whether such treatments alter lncRNA expression is not fully known. Searching the Interferome database (http://www.interferome.org/interferome/home.jspx, accessed on 1 July 2021), we observed that among the deregulated lncRNA, expression of 26 lncRNA increased and 20 lncRNA decreased at least in one time point by treatment with IFN-I and/or IFN-II. Changes in expression were dependent on the type of the IFN and the time of treatment (Tables S57 and S58). Expression of lncRNA was also varied in different experiments, possibly due to the use of different cell lines and conditions. The representative result is shown in Figure 7) and the detailed result is shown in Tables S22 and S23. The result obtained for the time points and type of IFN treatment reduced expression of EGOT, GABPB1-AS1, IDI2-AS1, LINC00312, LINC00662, MIAT, PVT1, SNHG7; expression of these lncRNA was increased in SARS-CoV-2 infected cells or in the tissue obtained from COVID-19 patients. Similarly, treatment with IFN increased the expression ST7-AS1; the expression of the lncRNA was decreased in COVID-19. In spite of variations in responses at different time points or from experiment to experiment, this analysis shows that several lncRNAs like MEG3, MALAT1, NEAT1, DANCR, DGCR5, DHRS4-AS1, and others could be regulated by INF.

### 3.2. Binding of Transcription Factors at the Putative Promoters of the Deregulated lncRNA

**Figure 8:**
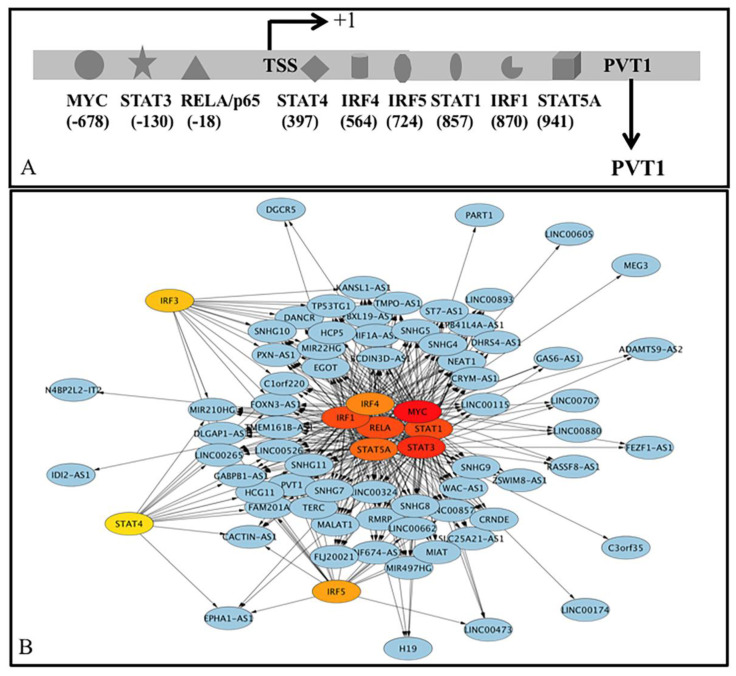
(**A**) (top panel): Binding of TFs to the putative promoter (−5 Kb to +1 Kb) of PVT1. Nine TFs IRF1 (binding positions 883 and 870 from the TSS), IRF4 (568, 564), IRF5 (724), MYC (933, 911, 862, 850, 832, 827, 824, 758, 711, 697, 455, 438, 434, 385, 365, 274, 191, 184, 163, 126, 98, 57, 45, 34, 31, 21, 20, 19, 3, −24, −54, −460, −617, −628, −678, −1454), RELA/p65 (974, 949, 932, 900, 894, 875, 863, 841, 831, 826, 806, 791, 679, 678, 677, 669, 657, 652, 652, 643, 633, 627, 626, 620, 612, 611, 603, 597, 596, 565, 341, 247, 18, −8), STAT1 (857,−4693), STAT3 (986, 844, 826, −130), STAT4 (397), STAT5A (941, 928) could bind to the putative promoter. Among the several binding sites observed from ChIPBase, only one site is shown in Figure. Positions indicate the distance from the transcription start site (TSS). Distances shown are not in scale. (**B**): (lower panel) Top ten interactions of TFs with lncRNAs represented by online software by Cytoscape. TF-Gene network being directional, the edges, connecting the nodes [TFs (shown in red and yellow filled ovals) and lncRNA (light blue)] are shown by arrows.

The mechanism(s) of deregulation of lncRNA is largely unknown. Given the importance of IFN-regulatory proteins (IRFs) and the JAK-STAT signaling pathway to induce expression of antiviral responses [109,110], we reported earlier that different IFN-regulatory proteins (IRFs), STAT1-STAT4, STA5A, and STAT6 could regulate the expression of several lncRNAs like MALAT1 and NEAT1 [34]. MYC interacts with MALAT1 and NEAT1 [111] and involves in influenza virus infection [112]. We observed earlier that MYC could regulate the expression of MALAT1 and NEAT1 by binding to the putative promoters [34]. In the present study, we extended the observations with additional deregulated lncRNAs. Besides, we included NF-kappa B RelA (RelA) as it has been implicated in viral replication as well as antiviral innate immune responses [113,114]. We searched for the binding of transcription factors IFR1-IFR5, IRF8, IRF9, STAT1-STAT4, STA5A, STAT6, MYC, and RELA at the putative promoters (−5 Kb to +1 kb) of the deregulated lncRNAs in ChIPBase v2.0 (http://rna.sysu.edu.cn/chipbase/, accessed on 1 July 2021) database as described earlier. This database catalogs ChIP-seq data in different cell lines at different conditions including treatment with different IFN as well as wild type MYC and MYC without having DNA binding activity and described earlier in detail [34]. Our analysis showed that at least one of the 15 TFs could bind to the putative promoters of 68 lncRNA (Tables S24 and S25). Binding of only one TF was observed for STAT3 (lncRNA C3orf35, LINC00174), IRF1 (IDI2-AS1 or N4BP2L2-IT2), and MYC (either LINC00605, MEG3, or PART1); in all other cases more than one TF could bind to the putative promoters of 61 lncRNA. Transcription factors IRF1 (2 binding sites), IRF4 (1), IRF5 (1), MYC (22), RELA (7), STAT1 (3), STAT3 (11), and STAT5A (3) could bind at the putative promoters (−5 Kb to +1 kb of transcription start site) of the lncRNA BCDIN3D-AS1. MYC, STAT3, RELA, IRF1, STAT1, STAT5A, IRF4, IRF5, IRF3, STAT4, STAT2, IRF2, NFKB2, IRF9 and STAT6 bind 63 (92.7%), 61 (89.7%), 53 (77.9%), 52 (76.5%), 50/68 (73.5%), 46 (67.7%), 40 (58.9%), 20 (29.4%), 17 (25.0%), 13 (19.1%), 10 (14.7%), 4 (5.9%), 5/68 (7.4%), 4/68 (5.9%) and 1 (1.5%) respectively. The same TF could activate or repress the expression of targets. All together 68 deregulated lncRNA 41 increased and 27 decreased) could be regulated at least by any one of the 15 TFs and target 3324 gene/protein (Tables S24–S27). A typical example of binding different TFs at −5 kb to +1 kb to PVT1 is shown in Figure 8A. Representation of all TF-lncRNA interaction using Cytoscape is shown in the Figure S4 and the top 10 interactions (Hub) is shown in Figure 8B.

#### 3.2.1. Deregulation of Protein Coding Genes in SARS-CoV-2 Infected Cells and Different Tissues from COVID-19 Patients

Differentially expressed PCG in cells expressing SARS-CoV-2 was determined from GSE147507, GSE152075, and GSE150392 [12,13,15,16] following methods described earlier [34]. Altogether, expressions of 2239 unique genes were increased, and 3252 unique genes were decreased in more than one experimental condition (data not shown).

#### 3.2.2. Regulation of PCG by Transcription Factors

**Figure 9:**
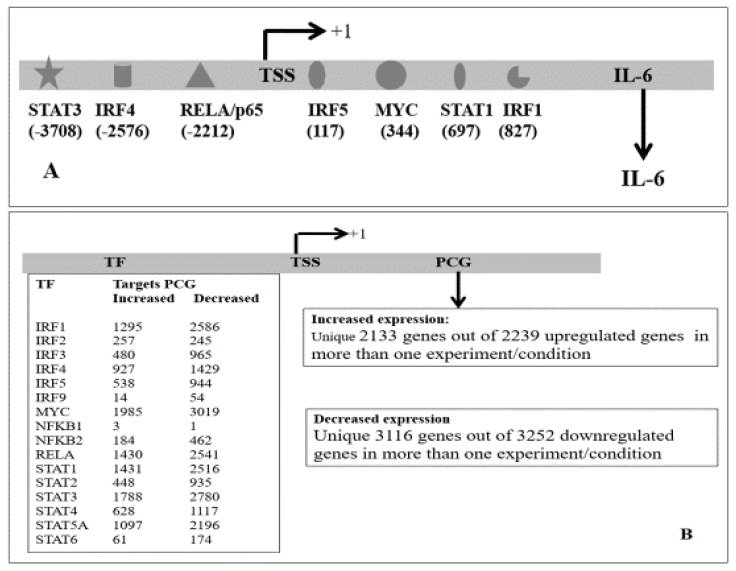
(**A**): Binding of different TFs at the putative promoter of IL-6 as obtained from ChIPBase. IRF1 (positions 994, 827, 713), IRF4 (−2576, −3246), IRF5 (117), MYC (878, 805, 738, 678, 676, 660, 646, 643, 636, 632, 613, 580, 494, 477, 344, −94, −2476), RELA (705, −2212), STAT1 (697, 490), STAT3 (987, 554, −3708) could bind to the putative promoter (−5Kb to +1Kb of the TSS). Only one of the binding sites of a TF is shown. Summary of the result obtained for all 16 TFs and 2133 upregulated genes and 3116 downregulated genes, where binding of at least one of 16 TFs was obtained is shown in (**B**). Detail result is shown in the Tables S28 and S29.

Among the PCGs that were increased in more than one experiment, protein class gene-specific transcriptional regulator (PC00264) as defined in PANTHER was higher (~15.6%) than that of the genes coded by the human genome (10. 6%) indicating that various transcription factors were deregulated in SARS-CoV-2 infected cells (data not shown). These TFs could contribute to the deregulated PCGs. Among the increased transcription factor IRFs, STATs, MYC, NFKB1, NFKB2, and NF-kappa B RELA have been implicated in viral infection [109,110,111,112,113,114]. We presented data in an earlier section that these TFs could regulate the expression of many lncRNAs as they have been identified to bind within the putative promoters of the lncRNA. We further tested whether PCGs could also be deregulated due to binding and regulation by these transcription factors. We downloaded the data from ChIPBase for binding of the TFs at the putative promoters of PCGs (data not shown) and combined it with the deregulated PCGs. The most putative promoters were occupied by more than one TFs and at multiple positions. We represented multiple positions of binding sites by a single TF (Table S28). It was observed that like lncRNA, MYC could bind to the putative promoters within −5 Kb to +1 Kb of most of the deregulated PCGs (91%), followed by STAT3 binding (83%). RelA/p65 and STAT1 both could bind 72% of the deregulated PCGs; IRF1, STAT5A, and IRF4 could bind to the promoters of 71%, 60%, and 43% deregulated genes respectively (Table S29). Thus, most of the deregulated PCGs in SARS-CoV-2 infected cells could be regulated by these transcription factors alone or in combination by binding to the putative promoters of the genes; uniquely, 2133 upregulated genes and 3116 downregulated genes in SARS-CoV-2 infected cells and in tissues from COVID-19 patients had at least one binding site of the above 16 TFs. Representative results of possible regulation of IL-6 by different TFs is shown Figure 9A and the summary of all result is shown in Figure 9B. The detail of the result is shown in Tables S28 and S29.

#### 3.2.3. Interferon Responsive PCG

As described above for lncRNA, we searched the Interferome database with 2239 increased and 3228 decreased genes. Similar to lncRNA, altered expression of PCG was dependent on the type of IFN, as well as the time of treatment. We observed that out of 1988 genes, expressions of 1674 genes were increased at least at one time point by IFN treatment among 2239 upregulated genes. Among the downregulated genes, expression of 2820 genes was altered by IFN treatment; expression of 2497 genes was decreased at least at one time point (Tables S30 and S31). This analysis showed dynamic changes of genes in response to the IFN treatment. Altered expressions of hundreds of gene in response IFN has been reported recently that involve in COVID-19 [121]

#### 3.2.4. Common Genes between lncRNA Interacting Proteins and Proteins Coded by the Deregulated Genes in SARS-CoV-2 Infected Cells and/or in Tissues from COVID-19 Patients

Comparing the deregulated lncRNA interacting gene/protein (3228) with downregulated (3252 unique genes) and upregulated genes (2239 unique gene), we observed that 299 upregulated genes and 810 downregulated genes are targets of the deregulated lncRNA (Figure 4C). LncRNA that interacts with DNA/promoter/chromatin might regulate the expression of genes at the transcriptional level; interaction with mRNA might alter the stability of mRNA. LncRNA that interacts with protein might not be captured in RNA sequencing data used in this study; in a small number of cases when lncRNA interacts with TFs and alter the stability of the TFs, it indirectly may alter the gene expression. However, if lncRNA interacts with TFs, spliceosome-associated proteins, or RNA-binding proteins, it might indirectly modify the mRNA level. Interaction of lncRNA directly or indirectly might alter the expression of the genes. Detailed results are shown in the Table S32.

### 3.3. Co-Regulation of PCG by Transcription Factors and lncRNAs

Levels of proteins are not only regulated by the TRFs by binding to the promoters but also at the post-transcriptional level by altering the stability of mRNA, during protein synthesis as well as post-translational level. LncRNA might be involved in all these stages (reviewed in Statello, Guo, Chen and Huarte [25], Walther and Schulte [26], Yao, Wang and Chen [27]). We have shown above that various TFs could regulate the expression of lncRNA and PCG by binding to the putative promoters and there were genes/proteins that were targets of lncRNA and also deregulated in SARS-CoV-2 infected cells/COVID-19. To find the deregulated PCGs that might be jointly regulated by the TFs and lncRNAs, forming over-represented FFLs, important motifs in gene regulatory networks and discussed in the introduction, we attempted to find out the joint targets (deregulated PCG) of TFs and lncRNA. Combining the results, we observed that altogether 15 TFs could bind and regulate the expression of 52 lncRNA and 1069 PCG. These 1069 genes or the proteins coded by these genes were targets of 52 deregulated lncRNA and might involve in the pathogenesis of SARS-CoV-2 infection. Detailed results are shown in the Table S33.

#### 3.3.1. Coregulation of PCGs by TF and lncRNA That Interacts with DNA/Promoters/Chromatin

**Figure 10:**
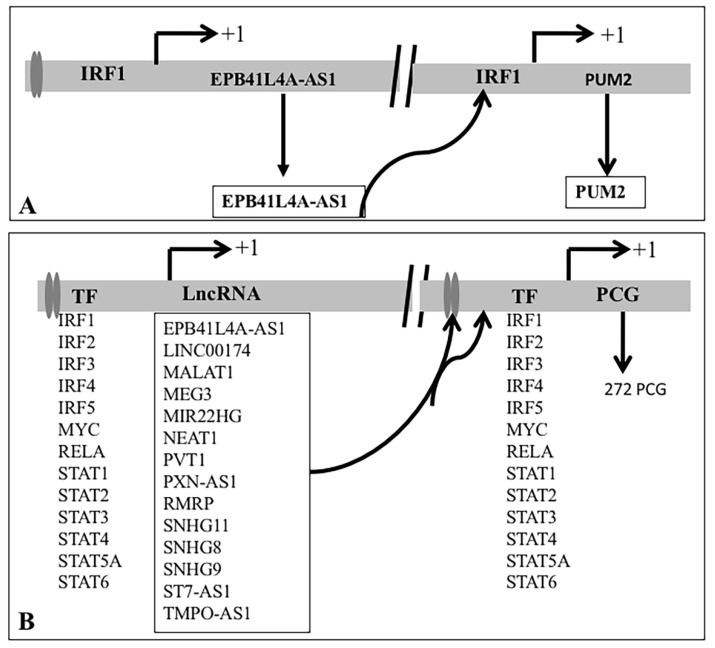
(**A**): Coregulation of PCG by IRF1 and EPB41L4A-AS1. IRF1 binds to DNA (filled horizontal lines) near lncRNA EPB41L4A-AS1 (5q22.1) at +756, +415, +254 of the TSS and PUM2 (2p24.1) at +959 of the TSS (http://rna.sysu.edu.cn/chipbase/, accessed on 1 July 2021). LncRNA EPB41L4A-AS1 also binds to DNA near the PUMA gene (NPInter v4.0, http://bigdata.ibp.ac.cn/npinter4, accessed on 1 July 2021). Solid vertical shapes represent the nucleosome. Vertical lines with a horizontal arrow represent the transcription star sites (TSS). (**B**): At least one of 13 transcription factors could bind to the DNA/chromatin of 14 lncRNA as shown could regulate 14 lncRNA and 272 PCG.

LncRNA binds to DNA/promoters/chromatin and modifies the transcription of the genes. We observed that any one of the 13 TFs could bind to the promoters of 14 lncRNA. It was further observed that altogether 272 deregulated PCGs were joint targets of at least one of the 13 TF and 14 DNA interacting lncRNAs. It was evident that MALAT1 and NEAT1 interacted with DNA near most of the 272 PCGs and were likely to modify (increase as well as decrease) the expression of the genes. PCG together with binding of IRF1 was likely to modify (increased as well as decreased). For example, PUM2 was a joint target of IRF1 and lncRNA EPB41L4A-AS1. IRF1 could bind to the putative promoter of the lncRNA EPB41L4A-AS1 and PUM2 as observed in the ChIPBase (Figure 10A). Expression of EPB41L4A-AS1 was increased in human induced pluripotent stem cell-derived cardiomyocytes (hiPSC-CMs) [15,97], cultured cells infected with SARS-CoV-2 [12] and PBMC of COVID-19 patients [14], and expression of PUM2 was also increased [12,15,97],). Thus, IRF1 regulated EPB41L4A-AS1 could bind to DNA/promoter of PUM2 and facilitate the expression of PUM2 by IRF1 (Figure 10A). Summary of the result for 14 lncRNA and 272 PCG that could be regulated by 13 TFs is shown in Figure 10B and detail is shown in the Table S34.

**Figure 11:**
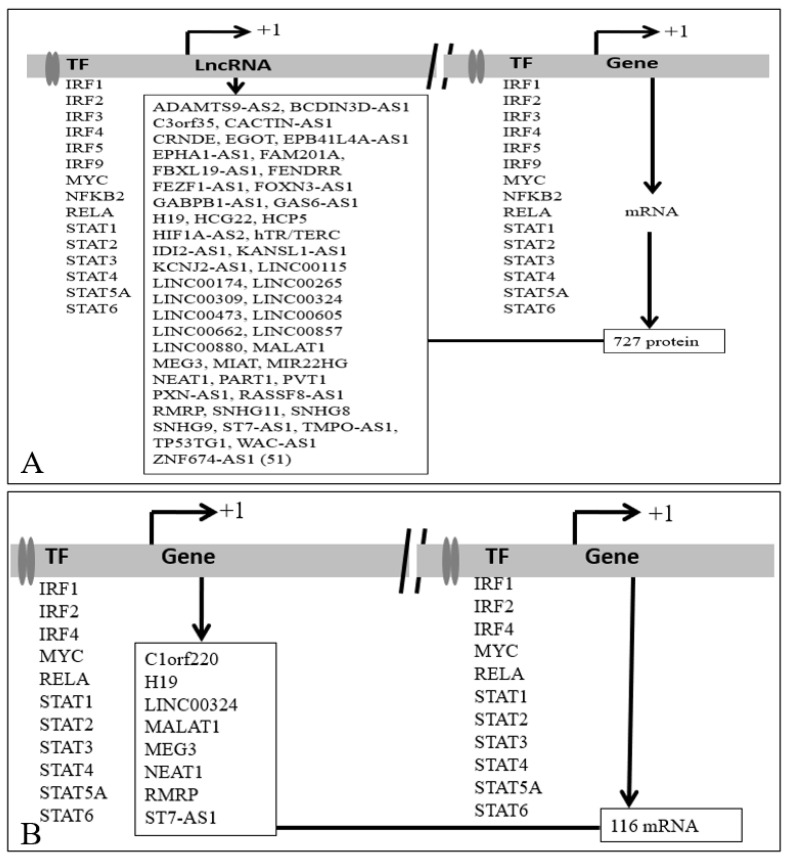
Summary of coregulated 727 PCG by 51 lncRNA (open box) that interacts (horizontal line joining lncRNA (within the box) with genes at protein and 15 TFs shown below DNA (filled rectangle) upstream to genes (**A**). Similarly, 116 mRNA co-regulated by 11 TFs and eight lncRNA. LncRNA and mRNA are regulated by 11 TFs (**B**).

Similar analysis with targets of lncRNAs at protein level revealed that 727 PCGs could be coregulated by 51 lncRNA and 15 TFs (Figure 11A). It was further observed that 116 mRNA that was the target of eight lncRNA could be regulated by 11 TFs (Figure 11B). Detailed result is shown in the Tables S38 and S43.

#### 3.3.2. Feedforward Motifs

**Figure 12:**
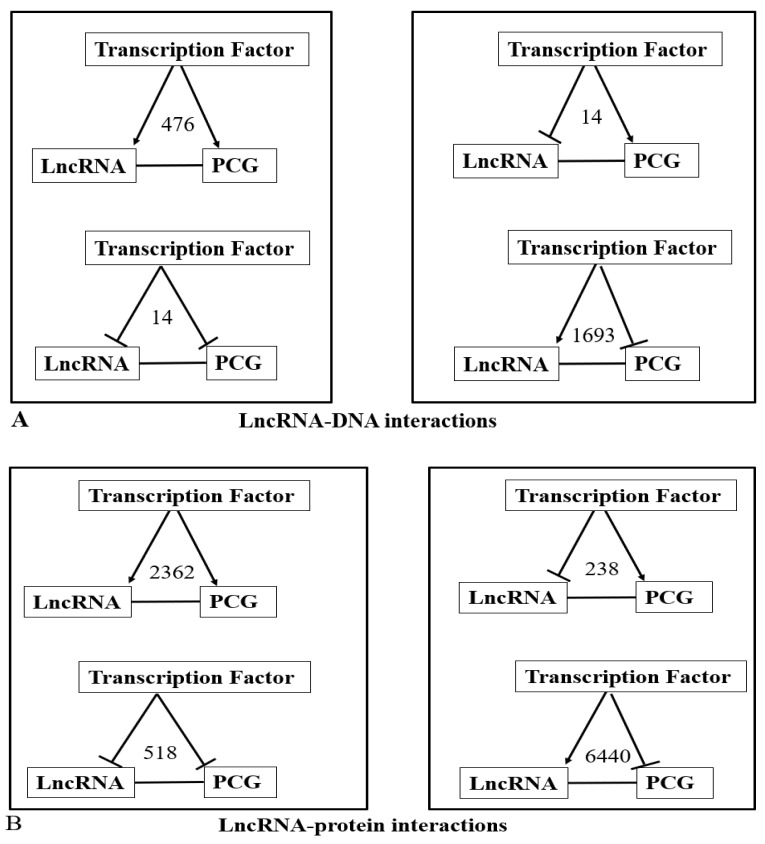

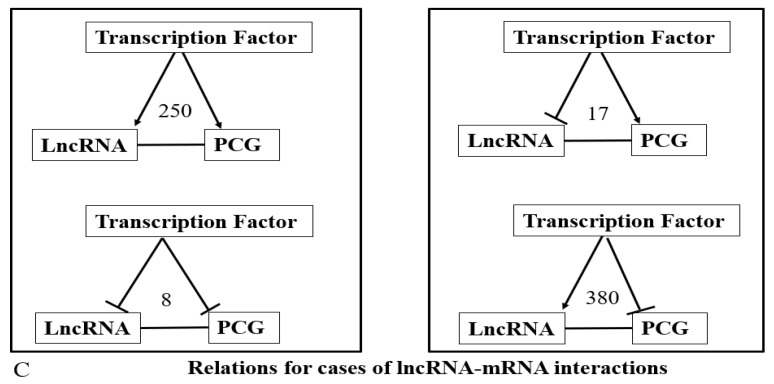
Relation of TF-lncRNA-PCG for lncRNA-DNA, lncRNA-protein and lncRNA-mRNA. Arrow represents activation, line with a vertical line (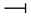) represents the repression of the target by TF and the horizontal lines represent interaction. Numbers represent the total relations in each category. (**A**–**C**) represent lncRNA-DNA, lncRNA-Protein and lncRNA-mRNA interactions respectively.

The exact effects of binding of TFs to the promoters of the lncRNA or PCGs were not known. Similarly, it is unknown whether the binding of TFs or interaction with lncRNA increase or decrease the targets. However, we know from our analysis that the combined effect of TFs was to increase or decrease the expression as the overall level of expression was known. From the above example of EPB41L4A-AS1 and PUM2 regulation by IRF1, it can reasonably be assumed that IRF1 activates the expression of both genes. Thus, when the expression of PCG, as well as lncRNA, increased we assume that TFs together could activate the expression of both the genes; for individual TFs this assumption might not be valid and require additional data. If the expression of PCG and lncRNA were in the same direction, we further assume that the effects of the interaction of lncRNA and PCG were to activate the PCG. With the above assumptions, we analyzed the common deregulated genes and targets of lncRNA (Tables S34, S38 and S43) and shown in detail in Tables S35–S37, S39–S42 and S44–S47. For example, among the DNA interacting lncRNAs, seven upregulated lncRNA (EPB41L4A-AS1, MALAT1, MEG3, NEAT1, PVT1, SNHG11, SNHG8) and 12 TFs (IRF1, IRF2, IRF4, IRF5, MYC, RELA, STAT1, STAT2, STAT3, STAT4, STAT5A, STAT6) could regulate increased expression of 70 PCGs and had 476 relations. Decreased expression of ELAVL1 could be regulated by decreased lncRNA PXN-AS1, SNHG9, TMPO-AS1, and TFs (IRF1, IRF3, MYC, RELA, STAT1, STAT5A) and had 14 relations. Decreased expression of SNHG9, ST7-AS1 and seven TFs (IRF1, IRF4, MYC, RELA, STAT1, STAT3, STAT5A) could jointly target HNRNPA1 and LSM11 that were decreased and had 14 relations. Increased expression of seven lncRNA (LINC00174, MALAT1, MIR22HG, NEAT1, RMRP, SNHG11, SNHG8) and 11 TFs (IRF1-IRF4, MYC, RELA, STAT1-STAT3, STAT5A, STAT6) could jointly target 201 PCG that was decreased in infected cells. The numbers of such motifs were 1693. The first two cases where expression of lncRNA and PCG was in the same directions represent coherent feedforward motif and functions as “failsafe” control. On the other hand, the latter two cases where expression of lncRNA and PCG was in opposite direction represent incoherent FFL and might have a “fine-tuning” function, maintaining the protein level in the appropriate functional range. This result is summarized in Figure 12A.

The result of a similar analysis for lncRNA that targets protein and mRNA is also shown in Figure 7B,C and the detailed result is shown in the Tables S39–S42 and S44–S47. The result shows that incoherent FFs, which maintain the protein level in the appropriate functional range were the predominant motif in joint targeting of the deregulated PCGs by lncRNA and TFs.

#### 3.3.3. Modification of Gene Expression of PCG by Knocking down or over Expression of lncRNA

The consequence of binding of lncRNA to DNA is likely to modulate the expression of the nearby gene; it remains mostly unknown whether such binding facilitates the expression or represses the expression. Similarly, binding of the lncRNA with mRNA or protein may stabilize or destabilize its interacting partners; in most of the cases the fate of such interaction is not known. There is a limited number of studies, where knockdown or overexpression of the lncRNA was followed by global gene expression studies by microarray or RNA sequencing, as well as low throughput assays (http://bio-annotation.cn/lncrna2target/index.jsp, accessed on 1 July 2021). We searched the database for the lncRNA which together with the TFs were involved in the coregulation of the PCG. Out of 52 lncRNA only seven lncRNA (FENDRR, hTR/TERC, LINC00473, MALAT1, MEG3, NEAT1 and PVT1) could modulate the expression of 135 PCG due to alteration of the lncRNA; knock down of MALAT1 and NEAT1 separately, in cultured cells could alter the expression of 121 coregulated genes. It was observed that both increased and decreased expression of PCGs were observed due to loss or gain of function of the lncRNA. Knockdown MYC in human promyelocytic leukemia cells resulted in down-regulation of PVT1 [122] supporting our analysis that MYC could bind to the putative promoter of PVT1. Besides, PVT1 knockdown by RNA interference led to suppression of the MYC protein and cell proliferation was inhibited [122]. Knockdown of PVT1 down-regulates the level of MYC protein in prostate cancer cell lines [123]. PVT1 interacts with MYC protein as cataloged in the NPInter database, thus the interaction of PVT1 with MYC protein is likely to stabilize the MYC protein, which in turn might increase the expression of PVT1. Both MYC and PVT1 were increased SARS-CoV-2 infected cells in culture ([12], GSE147507), human induced pluripotent stem cell-derived cardiomyocytes (hiPSC-CMs) infected with SARS-CoV-2 [15,97] GSE150392). Thus, in SARS-CoV-2 infected cells, MYC might regulate the expression of PVT1 and there might be a positive feedback loop between PVT1 and MYC. The result of the analysis is shown in Tables S48 and S49.

#### 3.3.4. Biological Processes and Pathways Enriched with Coregulated PCGs

Enrichment analysis with the joint targets of TFs and deregulated lncRNA revealed that these targets were associated with 297 pathways; 71 pathways were significantly (adjusted *p* ≤ 0.05) enriched with 303 PCGs (Table S50). We further clubbed the significantly enriched pathways and observed that several (i) infectious disease pathways including Coronavirus disease pathway (hsa05171) (131 genes), (ii) protein synthesis related pathways (27 genes), (iii) cell death (45 genes), (iv) signaling pathways (130 genes), (vi) proteasomal degradation (24 genes), (vii) metabolism related pathways (34 genes) were significantly enriched. Besides pathways related to different diseases like (a) neurological diseases (76 genes), (ii) cancers (62 genes) and (iii) metabolic diseases (51 genes) were also significantly enriched (Table S51). It is to be noted that a gene may be associated with different pathways. Enrichment analysis with the common targets of TF and lncRNA showed that 3975 biological processes (BP) defined by Gene Ontology were associated with the genes; 170 BP was significantly (adjusted *p* ≤ 0.05) enriched with 598 PCG (Table S52). BP related to gene expression (282 genes), mRNA metabolic processes including splicing (135 genes), protein synthesis (135 genes), protein degradation (103 genes), cell cycle (58 genes), cell growth, and death by apoptosis (140 genes), cytokines (71 genes), autophagy (24 genes), mitochondrial processes (17 genes), viral transcription processes (9 genes) and others were significantly enriched with common targets of TFs and deregulated lncRNA (Table S53).

## 4. Discussions

In silico analysis of deregulated lncRNA in SARS-CoV-2, infected cells or tissues from COVID-19 patients provided comprehensive information of their target genes/proteins and mechanism of deregulation lncRNAs by IRFs, STATs, MYC, and RelA/p65. In addition, coregulation of altered PCG by lncRNA and TFs identified several feedforward loops. Joint targets of TF and lncRNA were enriched with pathways and biological processes associated with SARS-CoV-2 infection.

### 4.1. Deregulated lncRNA

Out of 66 deregulated lncRNA was observed in more than one study, expression of 17 lncRNA CRNDE, DANCR, DLEU1, EGOT, EPB41L4A-AS1, GAS6-AS1, HCP5, LINC00526, LINC00605, MALAT1, NEAT1, PVT1, SNHG32, SNHG5, SNHG8, SNHG9, TP53TG1 in the same direction has been reported by other investigators. Altered expression of 39 lncRNA was new and expression of 10 lncRNA was reported by other investigators but in opposite direction. Infection with diverse viruses modulates the expression of lncRNA including SARS-CoV-2 [29,30,31]. Increased expression of NEAT1, MALAT1, EGOT, PVT1, MIAT has been also observed in infection with other viruses (Table S1). These lncRNAs might thus be involved in COVID-19 pathogenesis.

### 4.2. Involvement of Deregulated lncRNA in the Pathogenesis of COVID-19

Direct evidence that lncRNA is involved in the pathogenesis of COVID-19 is not available. Bioinformatics analysis with interacting partners of deregulated lncRNA in SARS-CoV-2 infected cells and comparison with experimental evidence for other viral infections inferred possible relevance of deregulated lncRNA in COVID-19 [32,33,34,35,36,37,38,39]. In the present study, in silico analysis revealed further that lncRNA interacting PCGs (i) interacted with SARS-CoV-2 coded proteins, (ii) deregulated SARS-CoV-2 infected cells, (iii) were modulated by interferon treatment, and (iv) modulated SARS-CoV-2 infection identified in a CRISPR screen. For example, we observed that H19 interacted with transcriptional repressor CTCF. CTCF also interacted with viral non-structural protein nsp15, downregulated in cells infected with SARS-CoV-2 as well as in the lungs of COVID-19 patients, and decreased by IFN treatment and identified as a modulator of viral infection [106]. The role of CTCF in COVID-19 is not known. However, the binding of CTCF to the viral genome and host genome in response to infection by other viruses has been observed. Recently, the role of CTCF in human cytomegalovirus (HCMV) latent infection altering gene expression has been established [124]. This in silico analysis indicated that CTCF might be involved in COVID-19 pathogenesis and require further experimental validation.

Various viral proteins coded by SARS-CoV-2 are known to interact with host splicing complex and translation machinery. Inhibition of mRNA splicing suppressed by the interaction of viral proteins with proteins involved in splicing also suppresses the host IFN response in SARS-CoV-2 infected cells [125]. Interaction of viral proteins with translational machinery leads to overall inhibition of mRNA translation in infected cells [125,126,127]. Our observations that several deregulated lncRNA interacts with proteins involved in nucleic acid metabolism like splicing, and protein synthesis, thus indicated that deregulated lncRNA might modulate splicing and protein synthesis and contribute to the pathogenesis.

Host proteins bind with the SARS-CoV-2 genome, as well as the viral transcripts. RNA binding proteins like ZC3HAV1, TRIM25, and PARP12 have been shown to possess antiviral activity and pro-viral activity. LARP1 has antiviral activity and interacts with the SARS-CoV-2 RNAs [128]. Nucleic acid-binding protein CNBP and LARP1 restrict SARS-CoV-2 replication in infected cells. Many SARS-CoV-2 RNA interacting partners like PUM1, G3BP1, G3BP2, CAPRIN1, DDX3X are associated with the IFN response. Pharmacological inhibition of viral RNA interacting host protein PPIA, ATP1A1, and the ARP2/3 complex, suppressed viral replication in cell lines [129]. This result shows that host RNA binding proteins might interact with the SARS-CoV-2 genome or transcripts and modulate viral infection. Some of the targets of deregulated lncRNA observed in our analysis were also SARS-CoV-2 RNA interacting proteins [128,129]. For examples, PPIA interacts with LINC00707; MALAT1 interacts with ATP1A1, CSDE1; NEAT1 interacts with SHFL, EIF3D and TRIM25. SARS-CoV-2 RNA binding protein CAPRIN1 interacts with LINC00324, LINC00662, MALAT1, PXN-AS1, and SNHG8; DDX3X interacts with 19 deregulated lncRNA including MALAT1 and NEAT1. Thus, the interaction of lncRNA with RNA binding proteins might modulate the functions of those proteins. Given a large number of RNA binding proteins were targets of lncRNA, RNA binding protein might competitively bind with viral genome/transcriptome and lncRNA and modulate the viral responses.

Abnormal IFN response has consistently been observed in many studies in COVID-19 [3,12,14,17,18,19,20,21]. In our analysis, it was observed that several deregulated lncRNA, as well as many PCG, could be regulated by IFN treatments. Expressions of several lncRNAs are shown to be modulated by treatment with IFNs [8,130,131]. Some of the deregulated lncRNA and PCG thus could be regulated in response to abnormal IFN in COVID-19. Based on the observations in other viruses than SARS-CoV-2 (Table S1), we speculated that NEAT1 might be involved in viral replication, regulating the expression of antiviral genes including cytokines, such as IL-8 possibly by facilitating nuclear paraspeckles formation, PVT1 might be involved in the regulation of gene expression, EGOT might be involved in the regulation of expression of ISGs and promotion of viral replication, MALT1 might be involved in epigenetic modification of viral gene expression and MEG3 might be involved in modulating p38 MAPK pathway by modulating the expression of TLR4, TNFα and IL-8, NF-κB. In summary, we presented in silico evidence that deregulated lncRNA observed in our analysis might be involved in COVID-19 pathogenesis by interacting with genes/proteins.

### 4.3. Mechanism of Deregulation of lncRNA and PCG

IRF1, STAT1, STAT3, RELA, and MYC independently or in combination might regulate most of the deregulated lncRNA or PCG observed in the present study as the putative promoters of these lncRNA were occupied by these TFs, mostly at the multiple sites. The binding of IRF1 and STAT1 to the promoters was observed in cells treated with IFNs. The binding of MYC to the promoters was observed only in wild type MYC expressing cells but not in cells expressing mutant MYC devoid of DNA binding domain and described earlier [34]. It is interesting to mention that the expression of most of the TFs was increased in SARS-CoV-2 infected cells (Table S60). It is not precisely known whether such binding activates or represses the target genes. In addition, we observed that expression of 46 lncRNA, as well as 4808 PCG, could be modulated by IFN treatment as evident from the Interferome database.

In infected cells, the host lncRNAs might modulate IFN induction for antiviral response by interacting with different components in the IFN induction pathway (reviewed [8,132]). Deregulation of the IFN pathways either by inborn errors or the generation of autoantibodies against type I IFNs or deregulation IFN signaling pathways have been associated with COVID-19 pathogenesis [133,134,135]. JAK-STAT signaling pathway in response to viral infection, in general, is the important component of the interferon response of the host. Recruitment of IFN to its receptor complex activates the receptor-associated JAK kinases leading to tyrosine phosphorylation, dimerization, and activation of STAT proteins. Activated by phosphorylation, STAT proteins form homo-or heterodimers and translocate to the nucleus, and activate the expression of interferon-stimulated genes (ISGs) [136]. Subsequent disruption of the JAK-STAT possibly through interactions of viral proteins with the host proteins allows viral replication, growth, and viral pathogenesis. In severe cases of COVID-19 patients, such disruption may lead to excessive cytokine production and multiple organ damage [137]. It has been shown that STAT1 and STAT2 phosphorylation and nuclear translocation are inhibited in SARS-CoV-2 infected cells due to the interaction of Orf6 with nuclear pore complex Nup98- Rae1 [138]. Proteomic analysis of SARS-CoV-2-infected cells revealed that JAK1, a key signaling protein, acted upstream of STATs and downstream of IFN and other cytokines, like interleukin IL-2, IL-4, IL-6, and IL-7. Expression of Tyk2 and IFNAR1 was decreased in SARS-CoV-2 infected cells [139]. The role of MYC regulated gene in SARS-CoV-2 infection is not fully known. However, MYC has been implicated in the regulation of the development of memory CD8(+) T cells in response to viral infection [140]. Activation of MYC has been observed in IAV infection [112], as well as proposed to target deregulated genes in SARS-CoV-2 infected cells [81]. Analysis of SARS-CoV-2 infected lung transcriptomics data identifies miRNAs that could target MYC indicating a possible role of MYC in the pathogenesis of COVID-19 [141]. Hyperactivation of the NFκB pathway involves the survival, activation, and differentiation of innate immune cells inflammatory T cells and has been implicated in the pathogenesis of the severe/critical COVID-19 patients [77]. This result supports our observations that most deregulated lncRNA and PCG might be regulated by IRF1, STAT1, STAT3, RELA, and MYC and be involved in the pathogenesis of COVID-19.

### 4.4. Coregulation of Deregulated PCG by TF and lncRNA and Their Associations with Infection Relevant Pathways

We utilized multiple data sources to identify joint targets of TFs known to be associated with viral infection and deregulated lncRNA in SARS-CoV-2 infected cells and observed coherent and incoherent FFL motifs. In a coherent FFL, TF induces the expression of both PCG and lncRNA. Interaction of lnCRNA with PCG is assumed to be increased. Thus, lncRNA might maintain the levels of PCG high when TFs fail to increase the target PCG for the proper functioning of PCG in specific conditions. Thus, this condition could be considered as failsafe control. In incoherent FFL, lncRNA might have a fine-tuning control for maintenance of the protein level in the appropriate functional range when TFs had opposite effects on lncRNA and PCG, similar to that described for microRNA [92].

Joint targets of TF and lncRNA were significantly enriched with pathways related to infectious disease including COVID-19, protein synthesis, cell death, signaling, proteasomal degradation, and others. Biological processes related to mRNA metabolic processes including splicing, protein synthesis, protein degradation, cell cycle, cell growth, cell death by apoptosis, cytokines, autophagy, mitochondrial processes were also significantly enriched. Significant association of the deregulated joint targets with pathways in different virus infections including Coronavirus indicates that the joint targets might modify the common steps of viral infection in general. Altered cytokines are the hallmark of COVID-19 [137]. Interaction of viral proteins with pre-mRNA complex proteins suppresses global splicing and the host IFN response in infected cells [125]. Interaction of viral protein with translational machinery and inhibition of host protein synthesis have been observed [126,127,142]. Proteasomal degradation [143], cell cycle [144], apoptosis [145], mitochondrial dysfunctions through excessive production of reactive oxygen species and affecting inflammation and apoptosis [146,147] have been implicated in the pathogenesis of COVID-19. Alterations of various signaling pathways like phosphatidylinositol 3-kinase (PI3K)/protein kinase B (AKT), interferon, p38 mitogen-activated protein kinase (MAPK), epidermal growth factor receptor (EGFR), and nuclear factor kappa-light-chain-enhancer of activated B cells (NF-κB) signaling pathways have been implicated in SARS-CoV and MERS-CoV. These signaling pathways are involved in antagonizing the host antiviral response and are vital for viral replication, entry, propagation, and apoptosis [148,149,150,151]. Common pathways enriched and known to be associated with viral pathogenesis indicate that the joint targets of TF and lncRNA might also be involved in the disease pathogenesis (for detailed enriched pathways and their possible relevance in viral infection please see Supplementary Sections S4.1.1–S4.1.5.

## 5. Limitations

The results presented here from in silico analysis to identify the deregulated expression of genes from largescale published RNA sequencing data requires further validation in low throughput assays. The binding of TFs to the putative promoters and regulation of the expression might be tissue-specific; we have used data available in cultured cells; thus, might differ in different affected tissues. The binding of TFs to the promoters and expression of the targets are dynamic, vary at different times after infection, and are not reflected in this analysis. Platforms used as well as the time after infection used in different studies might differ the result.

## 6. Conclusions

Targets of deregulated lncRNA might be involved in the pathogenesis of COVID-19 as the interacting partners could also interact with SARS-CoV-2 coded proteins deregulated in COVID-19; the expression is altered in response to IFN and modulates SARS-CoV-2 infection as identified in the CRISPR screen. Besides, deregulated PCG and lncRNA could be regulated by TFs, known to involve in SARS-CoV-2 infection. Co-regulation of PCG by TFs and lncRNA might play an important role in SARS-CoV-2 infected cells. Once validated in hypothesis-based studies, complex regulatory interactions of TFs and lncRNA shown here would not only help in understanding the intricate mechanisms of the disease but also new targets of intervention.

## Figures and Tables

**Table 1:** Altered expression of lncRNAs in cells transfected with SARS-CoV-2 infected cells or tissues from COVID-19 patients in more than one study.

Item	LncRNA (No)
Increased in the present study	ADAMTS9-AS2, BCDIN3D-AS1, C2orf27A, C3orf35, C6orf223, CACTIN-AS1, CRNDE, CRYM-AS1, EGOT, EPB41L4A-AS1, EPHA1-AS1, FBXL19-AS1, FEZF1-AS1, GABPB1-AS1, HCP5, IDI2-AS1, LINC00324, LINC00605, MALAT1, MIAT, MIR210HG, MIR22HG, MIR497HG, N4BP2L2-IT2, NEAT1, PVT1, RFPL3S, SNHG10, SNHG11, SNHG4, SNHG7, SNHG8, ZSWIM8-AS1 (33)
Increased in our earlier study [34]	HCG11, HIF1A-AS2, LINC00115, LINC00174, LINC00265, LINC00312, LINC00473, LINC00605, LINC00662, LINC00842, MALAT1, MEG3, MEG9, MIAT, NEAT1, RMRP, hTR/TERC, ZNF674-AS1 (20)
Decreased in the present study	ARIH2OS, C1orf220, DANCR, DGCR5, DHRS4-AS1, DLEU1, DLGAP1-AS1, FAM201A, FLJ20021, FOXN3-AS1, GAS6-AS1, H19, KANSL1-AS1, LINC00526, LINC00707, LINC00880, LINC00893, MIR1915HG, NBR2, NCBP2AS2, PXN-AS1, RASSF8-AS1, SLC22A18AS, SLC25A21-AS1, SNHG32, SNHG5, SNHG9, ST7-AS1, TMEM161B-AS1, TMEM99, TMPO-AS1, TP53TG1, and WAC-AS1 (33)
Decreased in our earlier study [34]	LINC00488, LINC00857, PART1, and TP53TG1 (4)

## Data Availability

Data of in silico analysis from publicly available resources is available in the Supplementary Files.

## Supplementary Materials

The following are available online at https://www.mdpi.com/article/10.3390/ncrna7040074/s1, Figure S1: Principles of regulation of PCG by TF, lncRNA and microRNA. Figure S2: Possible FFLs in TF-lncRNA joint targets network. Figure S3: Cytoscape representation of SARS-CoV-2 coded protein that interacts with deregulated lncRNA-interacting partners. Figure S4: Interaction of TFs with putative promoters of lncRNA visualized by Cytoscape. Figure S5: Cartoon representation of IFN synthesis by IRFs and finally induction of ISGs in collaboration with JAK-STAT signaling pathway to eliminate the viral infection. Table S1: Altered expression of long non-coding RNA by different viruses other than SARS-CoV-2. Table S2: Altered expression of long non-coding RNA in cells infected with SARS-CoV-2 and different tissues from COVID-19 patients. Table S3: Altered expression of long non-coding RNA in SARS-CoV-2 infected cells only in one experiment. Table S4: Altered expression of long non-coding RNA in more than one experiment. Table S5: Altered expression of long non-coding RNA in more than one experiment but in opposite direction. Table S6: Increased expression of lncRNA in more than one study. Table S7: Decreased expression of the lncRNA in the present study. Table S8: Altered expression of lncRNA in one experiments. Table S9: Comparison of deregulated lncRNA in the present study with the already published results. Table S10: Interacting partners of deregulated lncRNA in SARS-CoV-2 infected cells or COVID-19 patients (from Tables S6 and S7). Table S11: Interacting partners of decreased lncRNA in SARS-CoV-2 infected cells or COVID-19 patients (from Table S10). Table S12: Interacting partners of increased lncRNA in SARS-CoV-2 infected cells or COVID-19 patients (from Table S10). Table S13: Number of Interacting partner of deregulated lncRNA. Table S14: Protein classification of lncRNA interacting protein by PANTHER. Table S15: Proportion of protein class in different category. Table S16: SARS-CoV-2 coded protein interaction with host protein downladed from BioGrid. Table S17: Common lncRNA interacting protein/gene and viral protein interacting partners (from Tables S10 and S16). Table S18: LncRNA interacting protein with viral protein interacting partners (from Table S17). Table S19: Viral interacting proteins, their unique interacting host protein and their interacting lncRNA (from Table S17). Table S20: CRISPR screen for the involvement of host genes in modulation of SARS-CoV-2 infection from BioGRID. Table S21: LncRNA interacting protein that was identified in CRISPR assay. Table S22: Result of analysis of the deregulated lncRNA that were also altered in response to Interferon treatment. Table S23: Unique altered lncRNA that were also altered in response to IFN (from Table S22). Table S24: Binding of different TFs within -5Kb to +1Kb of the transcription start sites (ChIPBase v2.0). Table S25: Summary of TFs-LncRNA: number of binding sites at the putative promoters of the lnCRNA. Table S26: Possible regulation of lncRNA and their known targets. Table S27:Proportion of lncRNA regulated by different transcription factors. Table S28: Binding of different TFs at the putative promoters of altered PCG in SARS-CoV-2 infected cells/tissue from COVID-19 patients. Table S29: Proportion of PCG regulated by different transcription factors. Table S30: Altered expression of genes in response to IFN treatment among increased genes in SARS-CoV-2 infected cell. Table S31: Altered expression of genes in response to IFN treatment among decreased genes in SARS-CoV-2 infected cell. Table S32: Common deregulated PCG and lncRNA interacting gene/protein. Table S33: Coregulated PCG by lncRNA and TFs. Table S34: Coregulation of PCG by lncRNA that interacts with DNA and TFs (From Table S33). Table S35: Coregulation of decreased PCG by down regulated lncRNA that interacts with DNA and TFs (From Table S34). Table S36: Coregulation of increased PCG by down regulated lncRNA that interacts with DNA and TFs (From Table S34). Table S37: Coregulation of decreased PCG by up regulated lncRNA that interacts with DNA and TFs (From Table S34). Table S38: Coregulation of PCG by lncRNA that interacts with proteins and TFs (From Table S34–S37). Table S39: Coregulation of decreased PCG by down regulated lncRNA that interacts with protein and TFs (From Table S38). Table S40: Coregulation of increased PCG by down regulated lncRNA that interacts withprotein and TFs (From Table S38). Table S41: Coregulation of decreased PCG by up regulated lncRNA that interacts with protein and TFs (From Table S38). Table S42: Coregulation of increased PCG by up regulated lncRNA that interacts with protein and TFs (From Table S38). Table S43: Coregulation of PCG by lncRNA that interacts withmRNA and TFs (From S33). Table S44: Coregulation of decreased PCG by down regulated lncRNA that interacts with mRNA and TFs (From Table S43). Table S45: Coregulation of decreased PCG by up regulated lncRNA that interacts with mRNA and TFs (From Table S43). Table S46: Coregulation of increased PCG by down regulated lncRNA that interacts with protein and TFs (From Table S43). Table S47: Coregulation of increased PCG by up regulated lncRNA that interacts with protein and TFs (From S43). Table S48: Effects of knockdown/over expression of deregulated lncRNA level on PCG expression in highthroughput assays from LncRNA2target v3.0. Table S49: Effects of knockdown/over expression of deregulated lncRNA level on PCG expression observed in the present study (From Table S48). Table S50: Enriched pathways with joint targets of TFs and deregulated lncRNA. Table S51: Combined pathways in different categories. Table S52: Biological processes (BP) enriched with joint targets of TF and deregulated lncRNA in SARS-CoV-2 infected cells. Table S53: Combined biological processes in different categories. Table S54: Summary of studies for differential expression of lncRNA and protein coding genes. Table S55: Classification of lncRNA interacting proteins. Table S56: Representative enriched proteins of different classes among lncRNA interacting proteins. Table S57: Altered lncRNA expression in response to treatment with IFN1 or IFNII. Table S58: Representative result of altered expression of ncRNA in response to IFN treatment from Interferome v2.01. Table S59: Number of binding sites of different transcription factors at the putative promoters of PCG and lncRNA. Table S60: Altered expression of TFs used in this study.

## Abbreviations

Bronchoalveolar lavage fluid: BALF, Coronavirus disease 19: COVID-19, Endoplasmic reticulum: ER, Interferon: IFN, Interferon stimulated genes: ISG, Long non-coding RNA: lncRNA, Protein coding gene: PCG, Severe acute respiratory syndrome coronavirus 2: SARS-CoV-2, Supplementary Figure: SF, Supplementary Text Discussion Table: STDT, Supplementary Text Materials and Method: STMT, Supplementary Text Result Table: STRT, Supplementary Table for Introduction: STXI, Supplementary Table for Main Text: STX, Transcription factor: TF, Transcription factor: TF, Transcription start site: TSS.
