# Supplementary material for: Co-Regulation of Protein Coding Genes by Transcription Factor and Long Non-Coding RNA in SARS-CoV-2 Infected Cells: An In Silico Analysis
Source: Noncoding RNA. 2021 Nov 29;7(4):74. doi: 10.3390/ncrna7040074 (PMC8708613; doi:10.3390/ncrna7040074)
Supplement: Supplementary file 1 [file ncrna-07-00074-s001.zip › ncrna-1444146-supplementary-for proof final-proof done.pdf]

non-coding RNA

MDPI

Supplementary

S1. Supplementary Text Introduction (STI)

S1.1. Mechanism of Action of lncRNA in Viral Infection (in Connection with Section 1.2.2.4 in the Main Text.

S1.1.1. STI 1.1 Role of lncRNA in Viral Replication and Growth

The involvement of deregulated lncRNAs in antiviral activity by modulating viral replication and growth has been reviewed recently [1–3]. Interferon (IFN) treatments in vitro alter expressions of several lncRNAs and conversely several lncRNA have been shown to modulate IFN. Interferon (IFN) stimulated lncRNA ISR [4], MIR155HG [5], IVRPIE [6] suppress IAV replication and growth while LOC100506319/ LINC01988/ LncRNA-PAAN [7], TSPOAP1-AS1 [8], NRAV/DYNLL1-AS1 and PSMB8-AS1 [9] enhance IAV replication and growth as reviewed [2]. EGOT and CFAP58-DT/ lncITPRIP-1inhibit replication and growth of HCV [2]; NEAT1 inhibits replication and growth of HIV [10] and HTNV [11]. LncRNA32/LUARIS inhibits the replication of EMCV, HBV and HCV [12]. NRIR/lncCMPK2 suppresses HCV replication [13]. Overexpression of NeST/IFNG-AS1 inhibits clearance of Theiler's virus by increasing IFNG [2]. Increased expression of MIR155HG could enhance the level of INFB [5]. IVRPIE inhibits IAV replication by promoting IFN and ISGs expression, possibly by interacting with hnRNP U. IVRPIE regulates positively the expression of INFB and several ISGs such as IRF1, IFIT1, IFIT3, Mx1, ISG15, and IFI44L through histone modifications of target genes. These findings show that IVRPIE is a critical regulator of host antiviral response [6]. Various lncRNAs were identified in a loss of function screen to regulate IAV replication [7].

S1.1.2. STI1.2 Modulation of Viral Response by NEAT1 through Paraspeckle Formation

Increased expression of NEAT1 has been observed in cells infected with HIV, IAV, HTNV, HSV, Japanese encephalitis and rabies virus. HTNV infection in HUVEC cells with a reduced level of NEAT1 enhances HTNV replication while exogenous overexpression of NEAT1 effectively inhibited the replication. NEAT1 overexpression increased IFNB production and inhibited HTNV replication. A similar result was also observed in animals [11]. This result shows that NEAT1 affects HTNV viral replication through IFNB. Besides, NEAT1 has been shown to regulate the expression of IL8 by relocating SFPQ from its promoter and recruiting SFPQ into the paraspeckles [14]. Antiviral activity of NEAT1, observed in several experiments, could be mediated through the formation of paraspeckles, a class of membraneless subnuclear bodies, observed in the interchromatin space of mammalian cells. Paraspeckles are RNA–protein structures formed by the interaction between NEAT1, an indispensable structural component, and different classes of RNA and many RNA-binding proteins. Paraspeckles is induced by IAV, HSV [14] and HIV [10]. Paraspeckles could sequester paraspeckle-localizing proteins and RNA and modulates their functions outside the paraspeckles, thus acting as molecular sponges [15,16]. It has been revealed that NEAT1 may promote IFN responses by acting as positive feedback for RIG-I signaling. Increased NEAT1 by interacting with SFPQ, relocates SFPQ from the promoters of RIG-I and DDX60 to paraspeckles. Thus, NEAT1 removes the transcriptional inhibitory effects of SFPQ on RIG-I and DDX60, resulting in increased expression of transcriptional factor IRF7 which in turn induces the expression of IFN and NEAT1 [11]. NEAT1 may also activate IRF3 through the formation of a multi-subunit complex with HEXIM1. This NEAT1–HEXIM1 complex interacts with cGAS sensor and its partner PQBP1, and releases proteins from paraspeckle. Released proteins are recruited to STING and activate IRF3-producing type 1 IFN. These results indicate that NEAT1 has a critical role in the antiviral response of IFN through (i) RIG-I signaling and (ii) cGAS-STING-IRF3 pathway, reviewed in [1].

In summary, many deregulated lncRNA observed in different viral infections have been shown to modulate viral growth by regulating ISGs possibly due to interactions of lncRNA with host proteins. Contributions of lncRNA in alteration of host protein coding genes and diverse biological processes and pathways as observed in different other conditions [17,18] are not fully known.

S1.1.3. STI 1.3 Role of lncRNA in Innate Immunity in Response to Viral Infection

Infections with different viruses induce the host innate immunity system, the first line of defense against infection. In response to viral infection, the detection of viral components by the host immune system is mediated through a number of different receptors on and inside immune cells retinoic acid-inducible gene-I (RIG-I)-like receptors (RLRs), Toll-like receptors (TLRs) and NOD-like receptors (NLRs) and cyclic GMP-AMP synthase (cGAS). This in turns activates intracellular signaling cascades, leading to the secretion of type I IFNs and pro-inflammatory cytokines and chemokines. Following activation of the innate response, rapid induction of lncRNAs is mediated commonly through pro-inflammatory transcription factor NFκB. Deregulated lncRNAs modulate the expression of multiple inflammatory mediators. Many lncRNAs have been shown to interact with heterogeneous nuclear ribonucleoproteins, complexes that are implicated chromatin re-modelling, transcription process and translation. In addition, these lncRNAs have also been shown to interact with multiple other proteins involved in the regulation of chromatin remodelling, as well as those proteins involved in intracellular immune signaling, which include NF-κB. For example, lincRNA-COX2/Ptgs2os2, potential interacting partner of heterogeneous nuclear ribonucleoproteins hnRNP-A/B and hnRNP-A2/B1, regulated by NF-κB p65 and involved in chromatin re-modeling associated with the transcription of the late-primary inflammatory genes. Knockdown of lincRNA-COX2 inhibits CASP1 activation, decreases the secretion of IL-1β and TRIF cleavage, and results in TRIF-mediated autophagy. MALAT1, upregulated in response to LPS activation, has also been reported to regulate the innate immune response; knockdown of MALAT1 increased expression of inflammatory cytokines such as TNF-α, and IL-6. MALAT1 inhibits the activity of NF-κB by binding to the nuclear p65/p50 heterodimer. In response to pulmonary injury, MALAT1 regulates macrophage activation. Many other lncRNAs such as MIR3142HG, FIRRE (regulated by NF-κB), PACERR/PTGS2-AS1 (interacts NF-κB/p50 and modulates expression of the target genes of NF-κB p65/p50), THRIL, NRIR (regulated by IFN I) and others are involved in innate immune function. For detail, please see the review in [19].

Regulatory network: TFs-LncRNA-PCG (in connection with Section 1.3.1.)

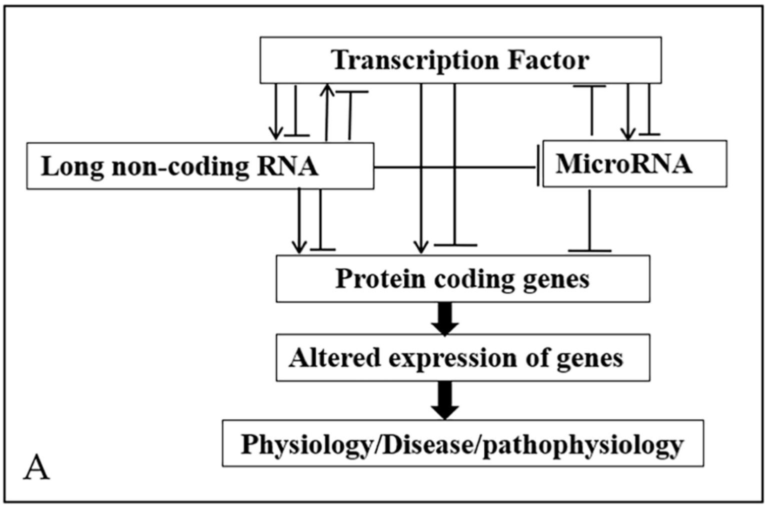

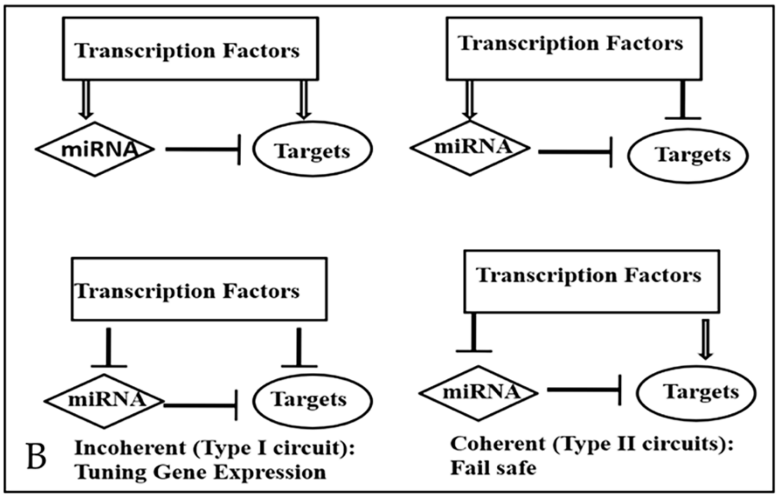

Figure S1. Principles of regulation of PCG by TF, lncRNA and microRNA (**A**) and coherent and incoherent feed forward motifs in TF-microRNA joint targets (**B**). Arrows represent activation while lines with a vertical line at the head represents repression.

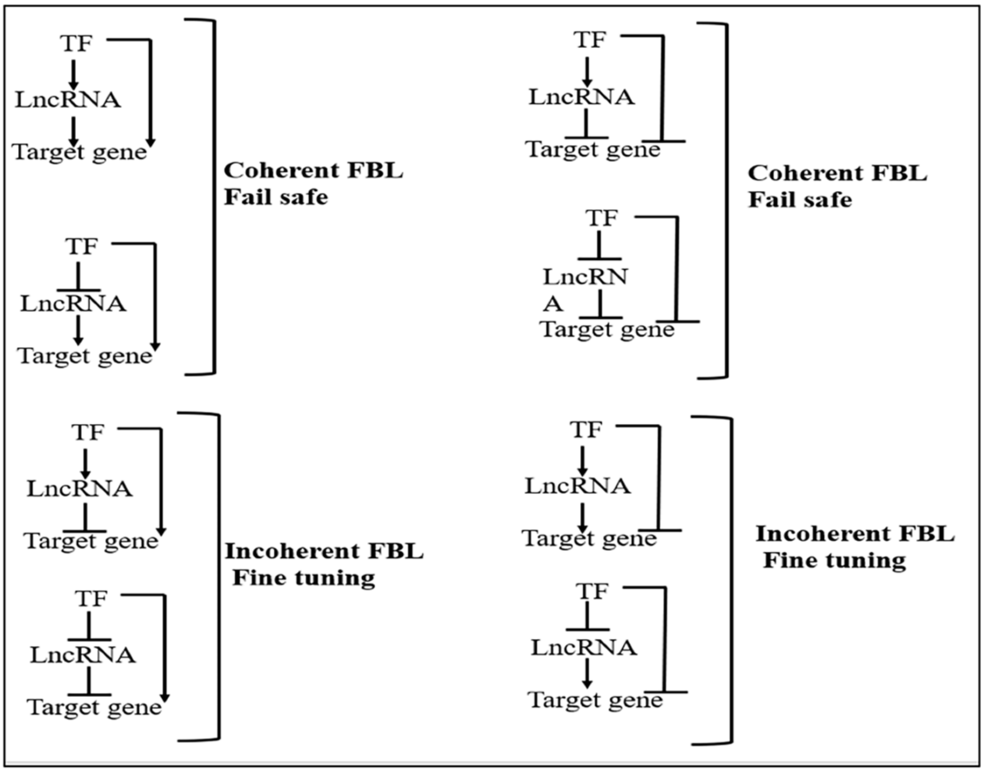

Figure S2. Possible FFLs in TF-lncRNA joint targets network. Possible function(s) of the FFL was assigned from the data in TF-microRNA targets as described [2]. Arrows represent activation while lines with a vertical line at the heads represent repression.

S2. Supplementary Text Material and Methods (STM)

Gene Expression Omnibus (GEO) Data Used (in Connection with Section 2.1)

S2.1.1. GSE147507

Different cell lines such as primary human lung epithelium (NHBE) cells, A549 cells (derived from human alveolar basal epithelial adenocarcinoma), Calu3 cells (derived from

human lung epithelial tumor) infected with SARS-CoV-2 (USA-WA1/2020), and lung tissue from COVID-19 patients were used and originally reported [21]. Detail of the conditions of infection and biological replicates, etc., has also been reported. All together 4 experimental conditions were used for differential expression of lncRNA: (i) expression in A549 cells infected with SARS-CoV-2 compared to expression in mock-transfected A549 cells, (ii) expression in A549 cells expressing exogenous ACE2 and infected with SARS-CoV-2 compared with that of in mock-transfected A549 cells, (iii) expression in A549 cells expressing exogenous ACE2 and infected with SARS-CoV-2 compared with that of in exogenous expressing ACE2 in A549 cells and (iv) expression in Calu3 cells infected with SARS-CoV-2 compared to expression in mock-transfected Calu3 cells [22]. There was a biological replicate of the condition in (iii). Besides, sequencing data for RNA samples from lungs tissues from COVID-19 patients and control were also analyzed. In the present study, we also analyzed PCGs using these cells and conditions.

S2.1.2. GSE150392

Human-induced pluripotent stem cell-derived cardiomyocytes (hiPSC-CMs) infected with SARS-CoV-2, a model system to examine the mechanisms of cardiomyocyte-specific infection by SARS-CoV-2, was used. These studies show that SARS-CoV-2 can infect hiPSC-CMs in vitro, establishing a model for elucidating the mechanisms of infection and potentially a cardiac-specific antiviral drug screening platform [23].

S2.1.3. GSE152075

RNA sequencing in nasopharyngeal swabs from 430 individuals with SARS-CoV-2 and 54 negative controls were carried out [24]

Table S54. Summary of studies for differential expression of lncRNA and protein coding genes.

Sample used	Type of analysis	Number of samples	Reference
Tissue culture model of primary human respiratory tract epithelial cells infected with SARS-CoV-2 (3D-airway model), COVID-19 patient (with co-morbidity) nasopharyngeal swab	Primary	20 subjects (10 Hi-VL and 10 Lo-VL)	[25]
Bronchoalveolar lavage fluid (BALF) and peripheral blood mononuclear cells (PBMC) samples from COVID-19 patients and healthy individuals	Reanalysis (CRA002390, BIG)	12 Chinese individuals (PBMC and BALF) and 3 healthy controls	[26]
PBMC	Reanalysis	COVID-19 admitted to ICU (n = 50), COVID-19 not admitted to ICU (n = 50), non-COVID-19 admitted to ICU (n = 16) and non-COVID-19 not admitted to ICU (n = 10),	[27]
PBMC	Primary	COVID-19 patients (*n* = 10) and healthy donors (*n* = 4) MSTRG tag	[28]
PBMC	Primary	17 severe, 12 non-severe patients and 10 healthy controls, severity determined by respiratory failure requiring mechanical	[29]

		ventilation; (2) signs of septic shock; (3) multiple organ failure requiring ICU admission	
Cell lines and BALF	Reanalysis	`	[30]
SARS-CoV-2 infected cells	Reanalysis	GSE147507	[22]

S3. Supplementary Text Result (STR)

S3.1 Protein Classes of the lncRNA-Interacting Partners (in Connection with Section 3.1.2 in the Main Text).

Protein classification of the lncRNA-interacting proteins using PANTHER (http://pantherdb.org/geneListAnalysis.do, 1 july 2021) revealed that targets of deregulated lncRNA include many proteins/genes classified as gene-specific transcriptional regulator (PC00264), such as CEBPB, ETS2, GLI4, HOXB7, HOXB8, IRF9, JUNB, KLF10, KLF15, (target of NEAT1), IRF2, JUND, MEF2D, STAT1 (target of MALAT1), HSF1, HSF4, IRF3, RELA/p65, STAT6 (target of MALAT1 and NEAT1), E2F1 (target of H19 and hTR/TERC), MYC (target of H19, hTR/TERC, MALAT1, NEAT1, and PVT1), TP53 (target of H19, TP53TG1, MEG3, NEAT1, and PVT1), and many others. Many genes/proteins associated with RNA metabolism such as splicing were also targets of the deregulated lncRNA. For example, splicing factor U2AF 35 kDa subunit gene U2AF1 (target of FAM201A, FOXN3-AS1, PART1, PXN-AS1, SNHG9, ST7-AS1, TMPO-AS1, CACTIN-AS1, CRNDE, EPB41L4A-AS1, FBXL19-AS1, GABPB1-AS1, HCP5, HIF1A-AS2, hTR/TERC, IDI2-AS1, LINC00174, LINC00265, LINC00324, LINC00662, MALAT1, MIR22HG, NEAT1, PVT1, RMRP, SNHG11, SNHG8), pre-mRNA-splicing factor SBCAS2, CD2BP2 (target of MALAT1 and NEAT1), HNRH1 (target of DLEU1, MALAT1, NEAT1, PVT1, and RMRP), and many others. Protein argonaute-1 AGO1/EIF2C1, associated with translation initiation, interacts with MALAT1, MIR22HG NEAT1, and PVT1. Protein ARGONAUTE-2 (AGO2) is also involved in translation initiation and interacts with DLEU1, FOXN3-AS1, GAS6-AS1, H19, LINC00880, RASSF8-AS1, SNHG9, ADAMTS9-AS2, CACTIN-AS1, CRNDE, EPB41L4A-AS1, EPHA1-AS1, GABPB1-AS1, HIF1A-AS2, IDI2-AS1, LINC00174, LINC00473, LINC00662, LINC00842, MALAT1, MEG3, MIT22HG, NEAT1, PVT1, and RMRP. Similarly, AGO3 and AGO4 also interact with many deregulated lncRNAs. Many other proteins such as EIF2B4, EIF2C, EIF2S3, EIF3B, EIF3C, EIF3CL, EIF3D, EIF3H, EIF4B, and others involved in translation initiation were targets of the deregulated lncRNA. Various proteins involved in translation elongation, such as EEF1A2, EEF1D, EEF1G, EEF2, and EEF2K, are targets of MALAT1 and/or NEAT1. This analysis is summarized in Table S55, S56; details in Tables S14 and S15 (in Excel) show that deregulated lncRNAs are associated with proteins involved in nucleic acid metabolisms such as splicing, transcription regulation, and protein synthesis. Such interaction of lncRNA with different proteins belonging to different classes may modulate the functions of those proteins.

Table S55. Classification of lncRNA-interacting proteins.

Protein Class	Proteins Coded by the Human Genome (12043/20595)	Proteins Coded by the Targets of Decreased lncRNA (164/233/246)	Proteins Coded by the Targets of Increased lncRNA (1932/3075/3179)
Nucleic acid metabolism protein (PC00171)	914/12043 (7.6%)	83/164 (50.6%)	259/1932 (13.4%)
DNA metabolism protein (PC00009)	215/910 (23.6%)	6/82 (7.3%)*	46/255 (18.0%)**
RNA metabolism protein (PC00031)	685/910 (75.3)	76/82 (92.7%)*	209/255 (82.0%)**
Gene-specific transcriptional regulator (PC00264)	1280/12043 (10.6%)	12/164 (7.3%)	218/1932 (11.3%)

Translational protein (PC00263)	335/12043 (2.8%)	20/164 (12.2%)	95/1932 (4.9%)
Chromatin/chromatin-binding, or -regulatory protein (PC00077)	284/12043 (2.4%)	7/164 (4.3%)	55/1932 (2.8%)

Out of 246 targets of decreased lncRNA, 233 genes were catalogued in PANTHER; 164 genes were classified into the protein class. *Out of 83 genes in the protein class nucleic acid metabolism protein (PC00171), 82 genes were classified into DNA metabolism protein (PC00009) (6 genes) and RNA metabolism protein (PC00031) (76 genes). **Out of 259 proteins in the class nucleic acid metabolism protein (PC00171), target of increased lncRNA, 255 proteins were further classified into DNA metabolism protein (PC00009) (46 genes) and RNA metabolism protein (PC00031) (209 genes).

Table S56 Representative enriched proteins of different classes among lncRNA-interacting proteins

Protein Class	lncRNA-Interacting Proteins	LncRNA
Gene-specific transcriptional regulator (PC00264)	CEBPB, ETS2, GLI4, HOXB7, HOXB8, IRF9, JUNB, KLF10, KLF15	NEAT1
	IRF2, JUND, MEF2D, STAT1	MALAT1
	HSF1, HSF4, IRF3, RELA/p65, STAT6	MALAT1 and NEAT1
	E2F1	H19 and hTR/TERC
	MYC	H19, hTR/TERC, MALAT1, NEAT1, and PVT1
	TP53	H19, TP53TG1, MEG3, NEAT1, and PVT1
RNA metabolism protein (PC00031)	U2AF 35 kDa subunit gene U2AF1 involves in splicing	FAM201A, FOXN3-AS1, PART1, PXN-AS1, SNHG9, ST7-AS1, TMPO-AS1, CACTIN-AS1, CRNDE, EPB41L4A-AS1, FBXL19-AS1, GABPB1-AS1, HCP5, HIF1A-AS2, hTR/TERC, IDI2-AS1, LINC00174, LINC00265, LINC00324, LINC00662, MALAT1, MIR22HG, NEAT1, PVT1, RMRP, SNHG11, SNHG8
	pre-mRNA-splicing factor SBCAS2, CD2BP2	MALAT1 and NEAT1
	Heterogeneous nuclear ribonucleoprotein H HNRNPH1	DLEU1, MALAT1, NEAT1, PVT1, and RMRP
Translational protein (PC00263)	AGO1/EIF2C1 [Translation initiation factor (PC00224)]	MALAT1, MIR22HG NEAT1, and PVT1
	AGO2/EIF2C2 [Translation initiation factor (PC00224)]	DLEU1, FOXN3-AS1, GAS6-AS1, H19, LINC00880, RASSF8-AS1, SNHG9, ADAMTS9-AS2, CACTIN-AS1, CRNDE, EPB41L4A-AS1, EPHA1-AS1, GABPB1-AS1, HIF1A-AS2, IDI2-AS1, LINC00174, LINC00473, LINC00662, LINC00842, MALAT1, MEG3, MIT22HG, NEAT1, PVT1, and RMRP
	Elongation factor 1-alpha 2 EEF1A2	MALAT1, NEAT1

S3.2 Interactions of SARS-CoV-2 Coded Proteins with Host Proteins (in Connection with Section 3.1.3 of the Main Text)

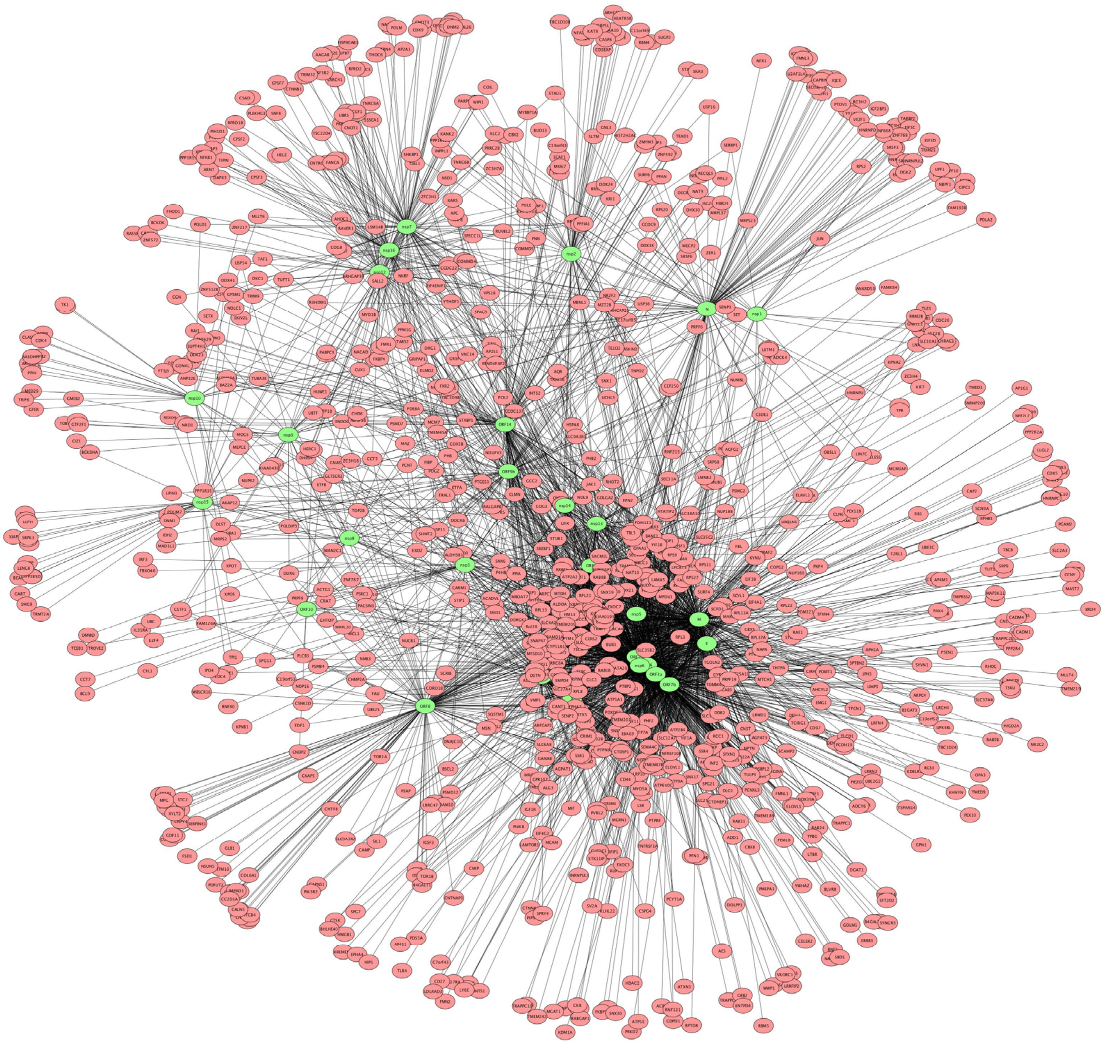

Figure S3. Cytoscape representation of SARS-CoV-2 coded protein (green) that interacts with deregulated lncRNA-interacting partners (magenta). Viral proteins that interact with lncRNA-interacting partners were obtained from the common genes/protein that interact with deregulated lncRNA and host proteins that interact with the viral proteins. Detailed results are shown in Tables S17–19.

S3.3 Possible Modulation of lncRNA Expression by IFN Treatment (Related to Section 3.1.5 in the Main Text)

Table S57. Altered lncRNA expression in response to treatment with IFN1 or IFNII

Expression increased for at least one time point	C2orf27A, C3orf35, C6orf223, CRNDE, EGOT, EPB41L4A-AS1, EPHA1-AS1, FBXL19-AS1, GABPB1-AS1, HCG11, HCP5, IDI2-AS1, LINC00265, LINC00312, LINC00324, LINC00662, MALAT1, MEG3, MIAT, MIR22HG, NEAT1, PVT1, SNHG10, SNHG11, SNHG7, ZNF674-AS1 (26)
Expression decreased for at least one time point	DGCR5, DHRS4-AS1, DLEU1, DLGAP1-AS1, FOXN3-AS1, H19, KANSL1-AS1, LINC00526, PART1, PXN-AS1, SLC22A18AS, SNHG5, SNHG9, ST7-AS1, TMEM161B-AS1, TMEM99, TMPO-AS1, and TP53TG1 (20)

Table S58: Representative result of altered expression of ncRNA in response to IFN treatment from Interferome database.

LncRNA	Interferon Type	Time of Treatment	Alteration
Increased expression of the lncRNA in COVID-19			

MEG3	Type 1	12h	Increased
	Type 1	72h	Decreased
	Type II	24h	Increased
MALAT1	Type 1	1.5h	Increased
	TYP2 II	8h	Increased
	TYP2 II	18h	Increased
NEAT1	Type 1	5h	Increased
	TYP2 II	18h	Decreased
	TYP2 II	24h	Increased
PVT1	Type II	24h	Decreased
EGOT	Type I	24h	Decreased
Decreased expression of the lncRNA in COVID-19			
DANCR	Type I	6, 12, 18,48, 72, 96 h	Decreased
	Type II	20, 24, 72, 96 h	Decreased
DGCR5	Type I	72h	Decreased
	Type II	24h	Increased
DHRS4-AS1	Type I	1.5h, 16h	Decreased
	Type II	1.5h	Decreased
	Type II	24h	Decreased/Increased

Table S59. Number of binding sites of different transcription factors at the putative promoters of PCG and lncRNA.

Transcription Factors	No of Target Genes (-5Kb to +1kb) in SARS-CoV-2-Infected Cells			
	Protein Coding Gene		LncRNA	
	Increased (2239)	Decreased (3252)	Increased (41/47)	Decreased (27/36)
IRF1	1295	2586	32	20
IRF2	257	245	3	2
IRF3	480	965	8	9
IRF4	927	1429	25	15
IRF5	538	944	13	7
IRF9	14	54	3	1
MYC	1985	3019	36	27
NFKB1	3	1	0	0
NFKB2	184	462	4	0
RELA	1430	2541	31	22
STAT1	1431	2516	31	19
STAT2	448	935	5	5
STAT3	1788	2780	36	25
STAT4	628	1117	8	5
STAT5A	1097	2196	28	18
STAT6	61	174	1	0

Unique 2133 upregulated genes and unique 3116 downregulated genes in SARS-CoV-2 infected cells and in tissues from COVID-19 patients had at least one binding sites of the above 16 TRFs. Out of 22 decreased expressions of lncRNA DLEU1, LINC00488, MIR1915HG, NBR2, and SNHG32 did not have binding of any one of the TFs studied. Out of 42 decreased expressions of lncRNA, C2orf27A, LINC00312, LINC00842, MEG9, and RFPL3S did not have binding of any one of the TFs studied.

S3.4 Transcription Factor–lncRNA Promoter Interactions (Related to Section 3.2 of Main Text)

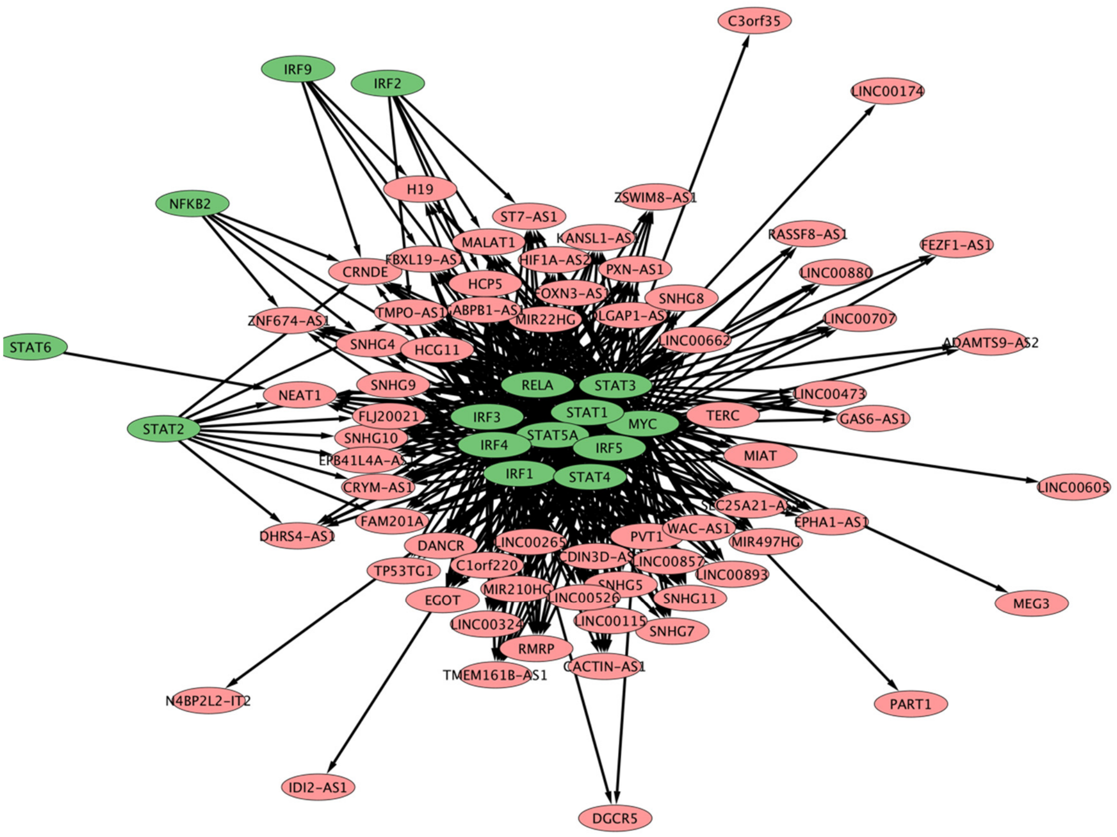

Figure S4. Interaction of TFs with putative promoters of lncRNA visualized by Cytoscape. TF-Gene network being directional, the edges, connecting the nodes (TFs (shown in green filled ovals) and lncRNA (red)), are shown by arrows.

S4. Supplementary Text Discussion (STD)

STD1: Altered expression of the TFs in cells infected with SARS-CoV-2 or in tissues from COVID-19 patients (related to discussion section "Mechanism of deregulation of lncRNA and PCG" in the main text)

Increased expression the TFs used observed in at least two independent studies, except for STAT3, which was increased only in one experiment**.**

Table S60. Altered expression of TFs used in this study.

Transcription factor	Altered	Condition	Reference
IRF1	Increased	Cell lines and hiPSC cardiomyocytes, hepatocytes, pancreatic cells infected with SARS-CoV-2, PBMC, nasopharyngeal swabs of patients	GSE147507, GSE150392*, GSE152075**
IRF2	Increased	Lung COVID-19, cell lines, hiPSC cardiomyocytes (hiPSC-CMs)	GSE150392, GSE147507
IRF3	Decreased	Cell lines infected with SARS-CoV-2	GSE147507
IRF4	Increased	Cell lines infected with SARS-CoV-2 in more than one experiment	GSE147507
IRF5	Increased	Cell lines infected with SARS-CoV-2, hiPSC cardiomyocytes	GSE147507, GSE150392
IRF6	Decreased	hiPSC cardiomyocytes and hepatocytes	GSE150392
	Increased	Cell lines infected with SARS-CoV-2	in GSE147507

IRF7	Increased	Nasopharyngeal swabs of patients, Cell lines and hiPSC cardiomyocytes infected with SARS-COV-2	GSE147507, GSE150392 GSE152075
IRF8	Increased	Cell lines and hiPSC cardiomyocytes infected with SARS-COV-2	GSE147507 GSE150392
IRF9	Increased	Cell lines infected with SARS-CoC-2 and lung of COVID-19	GSE147507
STAT1	Increased	Cell lines and hiPSC cardiomyocytes infected with SARS-COV-2, lung tissue and nasopharyngeal swabs of COVID-19 patients	GSE147507 GSE150392 GSE152075
STAT2	Increased	Cell lines and hiPSC cardiomyocytes infected with SARS-COV-2, nasopharyngeal swabs of COVID-19 patients	GSE147507 GSE150392 GSE152075
STAT3	Increased	hiPSC pancreatic cells (organoid)	[31]
STAT4	Increased	Cells infected with SARS-CoV-2, lung and nasopharyngeal swabs and PBMC of COVID-19 patients	GSE147507, GSE152075
	Decreased	hiPSC cardiomyocytes infected with SARS-COV-2	GSE150392
STAT5A	Increased	Cell lines infected with SARS-CoV-2	GSE147507
STAT5B	Increased	Cell lines infected with SARS-CoV-2	GSE147507
STAT6	Decreased	Cell lines infected with SARS-CoV-2	GSE147507
NFKB1/p50	Increased	Cell lines hiPSC hepatocytes infected with SARS-CoV-2, in PBMC from COVID-19 patients	GSE147507 GSE150392
NFKB2/p100	Increased	Cell lines hiPSC hepatocytes infected with SARS-CoV-2, in PBMC from COVID-19 patients***	GSE147507 GSE150392
RELA/p65	Increased	Cell lines infected with SARS-CoV-2	GSE147507
MYC (c-Myc)	Increased	Cell lines infected with SARS-CoV-2, lung tissue from COVID-19 patients, human induced pluripotent stem cell-derived cardiomyocytes (hiPSC-CMs) infected with SARS-CoV-2	GSE147507, GSE150392
	Decreased	Nasopharyngeal swabs	GSE152075

* Pluripotent stem cell-derived cardiomyocytes (hiPSC-CMs) infected with SARS-COV-2. **Nasopharyngeal swabs. ***PBMC [32]..

STD2: Description of enriched pathways with the joint targets of TFs and lncRNA and their possible involvement in viral infection (related to discussion section "Coregulation of deregulated PCG by TF and lncRNA and their associations with infection relevant pathways" in the main text).

Diverse biological pathways described in KEGG database and biological processes defined by Gene Ontology database were significantly enriched with common targets of TFs and deregulated lncRNA (Tables S50–53 as elaborated in the main text. Some of the significantly enriched pathways are described in detail.

Description of pathways

S4.1.1. IFN pathway

DNA/viral genomes/viral mRNAs or specific pathogen-associated molecular patterns (PAMPs) are recognized by host pattern recognition receptors (PRR) such as Toll-like receptors and RIG-I-like receptors and activate downstream signaling molecules such as such as adaptor proteins MAVS and MyD88, kinases TBK1 and IKK epsilon, and transcription factors IRF3 and NFκB. This in turn results in the production of type-I/III interferons and other cytokines. Multiplexed high-resolution mass spectrometry based

proteomic analysis of confirmed COVID-19 cases identified 7582 proteins. Significant up-regulation of interferon-mediated antiviral signaling was observed [33]. Simple model representation of IFN response is shown in Figure S5.

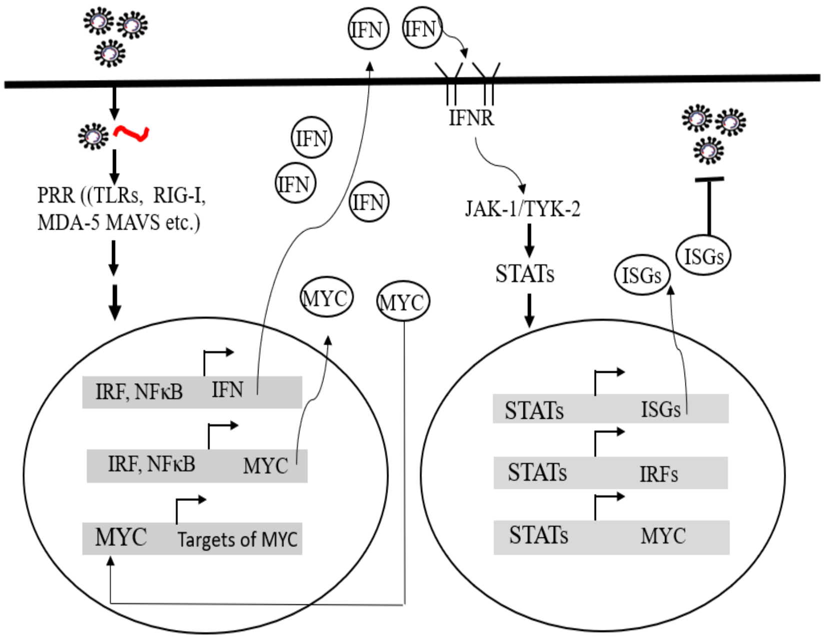

Figure S5. Cartoon representation of IFN synthesis by IRFs and finally induction of ISGs in collaboration with JAK-STAT signaling pathway to eliminate the viral infection. Red wavy line represents the viral RNA genome. To bypass the host antiviral response, the virus utilizes proteins coded by the virus to interfere with the pathway. Based on our analysis, MYC might be regulated by IRFs and RELA. Similarly, IRFs and MYC could also be regulated by STATs as binding of STATs were present at the putative promoters of the gene (Tables S28–29, in Excel). Abbreviations used in Figure SF3: ISGs: Interferon (IFN) stimulated genes, MAVs: mitochondrial antiviral signaling, MDA-5: melanoma differentiation-associated protein 5 (RIG-I like receptors), PRR: pattern recognition receptors, RIG-I: Retinoic acid-inducible gene I, TLR: Toll loke receptors.

In our analysis we also observed that some of these TFs could interact with several deregulated lncRNA. MYC could interact with H19, hTR/TERC, MALAT1, NEAT1, PVT1, SNHG7 interacted with MYC; NFKB1 interacted with hTR/TERC; IRF2 could interact MALAT1, IRF3 interacted with MALAT1, IRF9 interacted NEAT1, STAT1 could interact with MALAT1 and STAT6 interacted with MALAT1 and NEAT1. IRF2BP1, a corepressor of IRF2 interacts with IRF2 and the NFAT1-dependent transactivation of NFAT-responsive promoters [34]. Involvement of deregulated IFN pathways either by inborn errors or the generation of autoantibodies against type I IFNs or deregulation IFN signaling pathway has been associated with COVID-19 pathogenesis [35–37].

S4.1.2. JAK-STAT Signaling Pathway

JAK-STAT signaling pathway involves recruitment of IFN to its receptor complex and activation of the receptor associated JAK kinases leading to tyrosine phosphorylation, dimerization, and activation of STAT proteins. Activated by phosphorylation, STAT proteins form homo-or heterodimers and translocate to the nucleus and activates the expression of interferon stimulated genes (ISGs) [38]. JAK-STAT signaling pathway in response to viral infection, in general, is the important component of interferon response of the host. Subsequent disruption of the pathway possibly through interactions of viral proteins with the host proteins allows viral replication, growth and viral pathogenesis. In severe cases of COVID-19 patients, such disruption may lead to excessive cytokine production and multiple organ damage [39]. It has been shown that STAT1 and STAT2 phosphorylation and nuclear translocation are inhibited in SARS-CoV-2 infected cells due to interaction of

Orf6 with nuclear pore complex Nup98- Rae1[40]. Viral protein nsp6 binds TBK1 to suppress IRF3 phosphorylation, nsp13 binds and blocks TBK1 phosphorylation, and ORF6 binds importin KPNA2 to inhibit IRF3 nuclear translocation. It has further been shown that nsp1, nsp6, nsp13, ORF3a, M, ORF7a, and ORF7b suppress STAT1 and/or STAT2 phosphorylation, whereas ORF6 may inhibit a step downstream of STAT1/STAT2 phosphorylation [41]. SARS-CoV-2 coded nsp13, nsp14, nsp15 and orf6 potently repress primary interferon production and interferon signaling; orf6 shows the strongest suppression. It has further been shown that nsp13, nsp14, nsp15 and orf6 inhibit nuclear localization of IRF3 [42]. Proteomic analysis of SARS-CoV-2-infected cells revealed that JAK1, a key signaling protein acting upstream of STATs and downstream of IFN and other cytokines, such as interleukin IL-2, IL-4, IL-6, and IL-7. Expression of Tyk2 and IFNAR1 was also decreased in SARS-CoV-2 infected cells [43].

S4.1.3. TGF-β Pathway

Transforming growth factor-beta (TGFβ/TGFB) has been proposed to play an important role in lung fibrosis. In severe COVID-19 patients, TGF-β might be involved in fluid homeostasis in the lung. Block of TGF-β has been proposed to be a target of treatment of COVID-19 patients [44]. Proteomics studies identified disruption of various pathways due to interaction of viral proteins with host proteins. For example, interaction of ORF8 with TGF-β might modulate the TGF-β pathway [45]. In COVID-19 patients both dysregulated JAK/STAT pathway [46] may play an important role of TGF-β/Smad pathway [47]. Proteins involved in fibrosis, such as collagens, proteoglycans, integrins, the connective tissue growth factor, MMPs are activated by the TGF-β pathway and might also contribute to the pathogenesis of COVID-19. Activated TGF-β pathway has been observed in COVID-19 patients and may contribute to the lung fibrosis [48].

Tumor necrosis factor (TNF), an important cytokine can induce different intracellular signal pathways such as NF-kappa B pathway, the MAPK cascade, apoptosis and necroptosis as well as PI3K-dependent NF-kappa B pathway and JNK pathway leading to survival cell survival as well as inflammation and immunity. Various disrupted signaling pathways including TNF signaling pathway have been proposed to be targets of intervention in COVID-19 [49].

S4.1.4WNT/β-Catenin Pathway

This pathway is upregulated in severe sepsis-induced acute lung injury and sepsis mouse models and plays a significant role in fibrosis and inflammation. Various participating proteins in this pathway such as β-catenin are increased in COVID-19 patients and proposed to be targets of treatment [50] .

Endothelial activation and dysfunction have been implicated in pathogenesis of COVID-19 by promoting pro-coagulative state, inducing endothelial inflammation, and mediating leukocyte infiltration as reviewed [51].

VEGF level was positively correlated with viral titers [52] and considered to be a biomarker for the progression of COVID-19 [53].

S4.1.5Other Signaling Pathways

Host protein interacting partners of SARS-CoV-2-coded proteins might alter diverse pathways. These pathways include various antiviral immune response pathways such as HIF1 signaling, autophagy, RIG-I signaling, Toll-like receptor signaling, fatty acid oxidation/degradation, and IL-17 signaling, NF-kappaB signaling, PI3K-Akt signaling, MAPK signaling, and the AGE-RAGE signaling pathway. These pathways are involved in antagonizing the host antiviral response and are vital for viral replication, entry, propagation, and apoptosis. Coronaviruses manipulate the molecular function of signaling pathways, and this kind of interaction between the host cell and the virus might be responsible for viral pathogenesis. Changes in similar pathways in SARS-CoV-2-infected cells have been

proposed [54]. Using cell models, MAPK pathway has been implicated in lung fibrosis observed in COVID-19 [55]. These pathways could be hijacked or suppressed by the viral proteins, resulting in improved viral survival and life cycle.

From the literature search, diverse signaling pathways were identified that modulate SARS-CoV-2-infected cells and/or COVID-19 patients and are involved in the pathogenesis. These pathways include MAPK signaling pathways, chemokine, cytokine signaling pathway, Akt/mTOR/HIF-1 pathway, and pathways related to lipid metabolism and autophagy. The interactions of viral proteins with the host proteins associated with these pathways play important roles in the modulation of these pathways [56]. The involvement of diverse signaling pathways, possibly due to changes in the expression of ~100 human kinases, in COVID-19 has been reviewed [57].

S4.1.5.1. Pathways Associated with Mitochondrial Dysfunctions

Mitochondria play an important role in the pathogenesis of COVID-19 through excessive production of reactive oxygen species and affecting inflammation and apoptosis [58,59]. The Warburg effect, the enhancement of glycolysis with lactate production in hypoxic condition, has been implicated in the activation of pro-inflammatory macrophages and cytotoxic immune cells against pathogens. The role of oxidative metabolism in macrophages in the anti-inflammatory response in severe COVID-19 disease through alteration of many factors such as chronic hypoxia, mitochondrial senescence, enzymatic dysfunctions or deregulations, AMPK and p53 inactivation has been reviewed [60]. Increased expression of TP53 and decreased expression of SIRT1were observed in PBMC of COVID-19 patients compared to that in healthy controls. It was concluded that the inflammatory environment, the dysregulated p53/SIRT1 axis and low expression of IL7R and BLNK, important genes for lymphocyte homeostasis and function, might impact cell survival and B cell signaling [61] . Highly enriched Nsp1-repressed genes such as COX, NUDFA, NUDFB, and NUDFS families enriched with mitochondrial function and metabolism [62].

S4.1.5.2. Pathways Associated with mRNA Splicing

Various viral proteins coded by SARS-CoV-2 interact with the host splicing complex. NSP16 interacts with the pre-mRNA recognition domains of the U1 and U2 snRNAs and suppresses global splicing. Such inhibition of mRNA splicing suppresses the host IFN response in infected cells [63]. NSP1 repressed genes were significantly enriched with pathways mRNA processing [62].

S4.1.5.3. Pathways Associated with Translation/Protein Synthesis

Inhibition of host protein synthesis could be one of the strategies viruses adopt to evade antiviral activities. Inhibition of translation could be achieved by inactivating the translational machinery through interaction of viral proteins. NSP1, NSP4, NSP8, NSP9, NSP12, NSP15, NSP16, ORF3b, N, and E bind to specific host RNAs. Proteins coded by the mRNAs that interact with viral proteins such as COPS5, EIF1, and RPS12 were related to protein translation, protein transport (ATP6V1G1, SLC25A6, and TOMM20), protein folding (HSPA5, HSPA6, and HSPA1B). Viral protein nsp1 interacts with 18S ribosomal RNA in the mRNA entry channel of the ribosome and leads to overall inhibition of mRNA translation in infected cells. NSP1-mediated global translational inhibition by NSP1 has been shown to suppress host IFN response. NSP8 and NSP9 bind to the 7SL RNA in the signal recognition particle and interfere with protein trafficking to the cell membrane upon infection [63]. NSP1 overexpression in human cells alters the transcription profile drastically in cultured cells. Such alteration was not observed with mutant NSP1. Enriched pathways in the top NSP1 repressed genes showed that the most significant Gene Ontology groups include functional annotation clusters of ribosomal proteins and translation-related processes, ribosomal RNA processing and translation. More than 70 genes such as RPS, RPL, MRPS, and MRPL family members involved in translation and

regulators of translation are strongly repressed upon introduction of wild-type NSP1. The repression effect on these genes is completely absent in the NSP1 mutant. NSP1 has also been shown to block host mRNA translation [62]. NSP1 is associated with the 40S subunit of ribosomal preinitiation complexes and inhibits translation [64].

S4.1.5.4. Cell Cycle

Cell cycle arrest in response to viral infection is an important strategy for the survival and growth of virus in the host cells. The role of cell cycle processes in SARS-CoV-2 infection is poorly known. Infections with several viruses including SARS-CoV induce cell cycle arrest that facilitates viral replication. In coronavirus infections, interactions of viral-coded proteins with proteins related to the cell cycle have been observed and reviewed [65]. For example, SARS-CoV-coded accessory proteins 3a and 7b inhibit Rb phosphorylation by limiting the expression of cyclin D3 (CCND3) and block the cell cycle at G0/G1 phase. N protein coded by SARS-CoV inhibits the activity of cyclin–CDK complex and restricts progression of the S phase. Exogenous expression of viral NSP1 gene repressed a large number of mitotic cell cycle genes, including members in the CDK, CDC, and CCNB families, components of the centrosome, the anaphase-promoting complex, and various kinases. Repressed genes were significantly enriched with cell cycle and cell division, consistent with the reduced cell viability phenotype observed in NSP1 over expressed cultured cells [62]. In our analysis, NEAT1-interacting partners ANAPC2, ANAPC5, AURKA, CDK4, CDK5RAP3 and others were enriched with cell cycle processes. Expression of these genes was decreased in SARS-CoV-2-infected cells (Table S53) indicating that the cell cycle was impaired in COVID-19.

S4.1.5.5. Apoptosis

Apoptosis during viral infections could be beneficial for the host but deleterious for the virus, whose replication and growth is blocked in the absence of a viable susceptible host cell. However, virus-induced apoptosis might also be harmful to the host, damaging immune cells that should protect it. Apoptosis has been detected in SARS-CoV-2-infected human airway epithelial cells [66], human alveolar organoids [67], cultured cells [68], lung biopsy specimens of patients with COVID-19 [69] and lungs of SARS-CoV2-infected hamsters [70], possibly through CASP8 activation [71]. Viral protein ORF3a induces the cleavage and activation of caspase-8. Lymphopenia has been observed in severe COVID-19 patients and could be due to cytokine storm, that might lead to a pro-inflammatory status which, in turn, induces lymphocytes apoptosis. Expression of CD95 is increased in circulating CD4+ and CD8+ lymphocytes of infected subjects compared to healthy controls and could contribute to increased apoptosis in lymphoid cells of SARS-CoV-2 patients. PBMCs from COVID-19 patients show that several genes related to apoptosis and P53 signaling were increased and contribute to enhanced apoptosis and lymphopenia observed in COVID-19 patients. Possible role of apoptosis and other forms of cell death such as necroptosis and pyroptosis in SARS-CoV-2 infection and infection with other coronaviruses has been reviewed [72].

S4.1.5.6. Proteasomal Degradation Pathway

The role of the ubiquitin–proteasome system (UPS) in different steps of the coronavirus infection has been shown to be in the viral entry as well as RNA synthesis and protein expression [73]. SARS-CoV-2-coded N protein interacts with 11S proteasomal activator PA28γ, which regulates the intracellular abundance of the protein and degrading the viral protein degraded by PA28γ-20S in vitro degradation assay. Furthermore, we have identified proteasome activator PA28γ as an nCoV N binding protein by co-immunoprecipitation assay. As a result of their interaction, nCoV N could be degraded by PA28γ-20S in vitro degradation assay. This was also demonstrated by blocking de novo protein synthesis with cycloheximide. The stability of nCoV N in PA28γ-knockout cells was greater

than in PA28γ wild-type cells. Notably, immunofluorescence staining revealed that knockout of the PA28γ gene in cells led to the transport of N from the nucleus to the cytoplasm. Overexpression of PA28γ enhanced proteolysis of N compared to that in PA28γ-N151Y cells containing a dominant-negative PA28γ mutation, which reduced this process. These results suggest that PA28γ binding is important in regulating 20S proteasome activity, which in turn regulates levels of the critical nucleocapsid protein N of SARS-CoV-2 [74]. NSP1 repressed genes were enriched ubiquitin/proteasome pathways [62].

Multiplexed high-resolution mass spectrometry based proteomic analysis of confirmed COVID-19 cases and negative controls identified the downregulation of several proteasomal subunits, E3 ubiquitin ligases, indicating a possible role of protein degradation pathway in COVID-19 [33].

Our analysis revealed that several targets such as PSMB4, PSMB5, PSMC3, PSMC5, PSMD4, PSMD9, PSMA7, UBE2I, UBE2J2, UBE2M, and UBE2S of increased NEAT1 and MALAT1 were associated with protein degradation pathways. Expression of these genes was decreased (Table S51). This result is similar to that reported using the proteomics approach [33].

References

1. Qiu, L.; Wang, T.; Tang, Q.; Li, G.; Wu, P.; Chen, K. Long Non-coding RNAs: Regulators of Viral Infection and the Interferon Antiviral Response. *Front. Microbiol.* **2018**, *9*, 1621–1621.
2. Liu, W.; Ding, C. Roles of LncRNAs in Viral Infections. *Front. Cell. Infect. Microbiol.* **2017**, *7*, 205.
3. Valadkhan, S.; Plasek, L.M. Long Non-Coding RNA-Mediated Regulation of the Interferon Response: A New Perspective on a Familiar Theme. *Pathog. Immun.* **2018**, *3*, 126–148.
4. Pan, Q.; Zhao, Z.; Liao, Y.; Chiu, S.-H.; Wang, S.; Chen, B.; Chen, N.; Chen, Y.; Chen, J.-L. Identification of an Interferon-Stimulated Long Noncoding RNA (LncRNA ISR) Involved in Regulation of Influenza A Virus Replication. *Int. J. Mol. Sci.* **2019**, *20*, 5118.
5. Maarouf, M.; Chen, B.; Chen, Y.; Wang, X.; Rai, K.R.; Zhao, Z.; Liu, S.; Li, Y.; Xiao, M.; Chen, J.L. Identification of lncRNA-155 encoded by MIR155HG as a novel regulator of innate immunity against influenza A virus infection. *Cell Microbiol.* **2019**, *21*, e13036.
6. Zhao, L.; Xia, M.; Wang, K.; Lai, C.; Fan, H.; Gu, H.; Yang, P.; Wang, X. A Long Non-coding RNA IVRPIE Promotes Host Antiviral Immune Responses Through Regulating Interferon β1 and ISG Expression. *Front. Microbiol.* **2020**, *11*, 260.
7. Wang, J.; Wang, Y.; Zhou, R.; Zhao, J.; Zhang, Y.; Yi, D.; Li, Q.; Zhou, J.; Guo, F.; Liang, C.; et al. Host Long Noncoding RNA lncRNA-PAAN Regulates the Replication of Influenza A Virus. *Viruses* **2018**, *10*. doi.: 10.3390/v10060330.
8. Wang, Q.; Zhang, D.; Feng, W.; Guo, Y.; Sun, X.; Zhang, M.; Guan, Z.; Duan, M. Long noncoding RNA TSPOAP1 antisense RNA 1 negatively modulates type I IFN signaling to facilitate influenza A virus replication. *J. Med. Virol.* **2019**. 10.1002/jmv.25483.
9. More, S.; Zhu, Z.; Lin, K.; Huang, C.; Pushparaj, S.; Liang, Y.; Sathiaseelan, R.; Yang, X.; Liu, L. Long non-coding RNA PSMB8-AS1 regulates influenza virus replication. *RNA Biol.* **2019**, *16*, 340–353.
10. Zhang, Q.; Chen, C.Y.; Yedavalli, V.S.; Jeang, K.T. NEAT1 long noncoding RNA and paraspeckle bodies modulate HIV-1 posttranscriptional expression. *MBio.* **2013**, *4*, e00596–e00512.
11. Ma, H.; Han, P.; Ye, W.; Chen, H.; Zheng, X.; Cheng, L.; Zhang, L.; Yu, L.; Wu, X.a.; Xu, Z.; et al. The Long Noncoding RNA NEAT1 Exerts Antihantaviral Effects by Acting as Positive Feedback for RIG-I Signaling. *J. virol.* **2017**, *91*, e02250–e02216.
12. Nishitsuji, H.; Ujino, S.; Yoshio, S.; Sugiyama, M.; Mizokami, M.; Kanto, T.; Shimotohno, K. Long noncoding RNA #32 contributes to antiviral responses by controlling interferon-stimulated gene expression. *Proc Natl Acad Sci U S A* **2016**, *113*, 10388–10393.
13. Kambara, H.; Niazi, F.; Kostadinova, L.; Moonka, D.K.; Siegel, C.T.; Post, A.B.; Carnero, E.; Barriocanal, M.; Fortes, P.; Anthony, D.D.; et al. Negative regulation of the interferon response by an interferon-induced long non-coding RNA. *Nucleic Acids Res.* **2014**, *42*, 10668–10680.
14. Imamura, K.; Imamachi, N.; Akizuki, G.; Kumakura, M.; Kawaguchi, A.; Nagata, K.; Kato, A.; Kawaguchi, Y.; Sato, H.; Yoneda, M.; et al. Long noncoding RNA NEAT1-dependent SFPQ relocation from promoter region to paraspeckle mediates IL8 expression upon immune stimuli. *Mol. Cell.* **2014**, *53*, 393–406.
15. Nakagawa, S.; Yamazaki, T.; Hirose, T. Molecular dissection of nuclear paraspeckles: Towards understanding the emerging world of the RNP milieu. *Open Biol.* **2018**, *8*. doi.: 10.1098/rsob.180150.
16. Fox, A.H.; Lamond, A.I. Paraspeckles. *Cold Spring Harb Perspect Biol.* **2010**, *2*, a000687.
17. Statello, L.; Guo, C.-J.; Chen, L.-L.; Huarte, M. Gene regulation by long non-coding RNAs and its biological functions. *Nat. Rev. Mol. Cell Biol.* **2021**, *22*, 96–118.
18. Yao, R.-W.; Wang, Y.; Chen, L.-L. Cellular functions of long noncoding RNAs. *Nat. Cell Biol.* **2019**, *21*, 542–551.

19. Hadjicharalambous, M.R.; Lindsay, M.A. Long Non-Coding RNAs and the Innate Immune Response. *ncRNA* **2019**, *5*. doi.:10.3390/ncrna5020034
20. Osella, M.; Bosia, C.; Corá, D.; Caselle, M. The role of incoherent microRNA-mediated feedforward loops in noise buffering. *PLoS Comput. Biol.* **2011**, *7*, e1001101.
21. Blanco-Melo, D.; Nilsson-Payant, B.E.; Liu, W.C.; Uhl, S.; Hoagland, D.; Møller, R.; Jordan, T.X.; Oishi, K.; Panis, M.; Sachs, D.; et al. Imbalanced Host Response to SARS-CoV-2 Drives Development of COVID-19. *Cell* **2020**, *181*, 1036–1045.
22. Laha, S.; Saha, C.; Dutta, S.; Basu, M.; Chatterjee, R.; Ghosh, S.; Bhattacharyya, N.P. In silico analysis of altered expression of long non-coding RNA in SARS-CoV-2 infected cells and their possible regulation by STAT1, STAT3 and interferon regulatory factors. *Heliyon* **2021**, *7*, e06395.
23. Sharma, A.; Garcia, G.; Arumugaswami, V.; Svendsen, C.N. Human iPSC-Derived Cardiomyocytes are Susceptible to SARS-CoV-2 Infection. *bioRxiv* 2021 in press.
24. Lieberman, N.A.P.; Peddu, V.; Xie, H.; Shrestha, L.; Huang, M.L.; Mears, M.C.; Cajimat, M.N.; Bente, D.A.; Shi, P.Y.; Bovier, F.; et al. In vivo antiviral host transcriptional response to SARS-CoV-2 by viral load, sex, and age. *PLoS Biol.* **2020**, *18*, e3000849.
25. Devadoss, D.; Acharya, A.; Manevski, M.; Pandey, K.; Borchert, G.M.; Nair, M.; Mirsaeidi, M.; Byrareddy, S.N.; Chand, H.S. Distinct Mucoinflammatory Phenotype and the Immunomodulatory Long Noncoding Transcripts Associated with SARS-CoV-2 Airway Infection. *medRxiv* **2021,** in press.
26. Moazzam-Jazi, M.; Lanjanian, H.; Maleknia, S.; Hedayati, M.; Daneshpour, M.S. Interplay between SARS-CoV-2 and human long non-coding RNAs. *J. Cell. Mol. Med.* **2021**, *25*, 5823–5827.
27. Shaath, H.; Alajez, N.M. Identification of PBMC-based molecular signature associational with COVID-19 disease severity. *Heliyon* **2021**, *7*, e06866.
28. Li, C.X.; Chen, J.; Lv, S.K.; Li, J.H.; Li, L.L.; Hu, X. Whole-Transcriptome RNA Sequencing Reveals Significant Differentially Expressed mRNAs, miRNAs, and lncRNAs and Related Regulating Biological Pathways in the Peripheral Blood of COVID-19 Patients. *Mediators Inflamm.* **2021**, *2021*, 6635925.
29. Cheng, J.; Zhou, X.; Feng, W.; Jia, M.; Zhang, X.; An, T.; Luan, M.; Pan, Y.; Zhang, S.; Zhou, Z.; et al. Risk stratification by long non-coding RNAs profiling in COVID-19 patients. *J. Cell. Mol. Med.* **2021**, *25*, 4753–4764.
30. Mukherjee, S.; Banerjee, B.; Karasik, D.; Frenkel-Morgenstern, M. mRNA-lncRNA Co-Expression Network Analysis Reveals the Role of lncRNAs in Immune Dysfunction during Severe SARS-CoV-2 Infection. *Viruses* **2021**, *13*.
31. Yang, L.; Han, Y.; Nilsson-Payant, B.E.; Gupta, V.; Wang, P.; Duan, X.; Tang, X.; Zhu, J.; Zhao, Z.; Jaffré, F.; et al. A Human Pluripotent Stem Cell-based Platform to Study SARS-CoV-2 Tropism and Model Virus Infection in Human Cells and Organoids. *Cell Stem Cell* **2020**, *27*, 125–136.e127.
32. Lee, J.S.; Park, S.; Jeong, H.W.; Ahn, J.Y.; Choi, S.J.; Lee, H.; Choi, B.; Nam, S.K.; Sa, M.; Kwon, J.S.; et al. Immunophenotyping of COVID-19 and influenza highlights the role of type I interferons in development of severe COVID-19. *Sci. Immunol.* **2020**, *5*. doi.: 10.1126/sciimmunol.abd1554
33. Vanderboom, P.M.; Mun, D.-G.; Madugundu, A.K.; Mangalaparthi, K.K.; Saraswat, M.; Garapati, K.; Chakraborty, R.; Ebihara, H.; Sun, J.; Pandey, A. Proteomic Signature of Host Response to SARS-CoV-2 Infection in the Nasopharynx. *Mol. Cell. Proteomics.* **2021**, *20*, 100134–100134.
34. Carneiro, F.R.G.; Ramalho-Oliveira, R.; Mognol, G.P.; Viola, J.P.B. Interferon regulatory factor 2 binding protein 2 is a new NFAT1 partner and represses its transcriptional activity. *Mol. Cell. Biol.* **2011**, *31*, 2889–2901.
35. Bastard, P.; Gervais, A.; Le Voyer, T.; Rosain, J.; Philippot, Q.; Manry, J.; Michailidis, E.; Hoffmann, H.H.; Eto, S.; Garcia-Prat, M.; et al. Autoantibodies neutralizing type I IFNs are present in ~4% of uninfected individuals over 70 years old and account for ~20% of COVID-19 deaths. *Sci. Immunol.* **2021**, *6*. doi.:10.1126/sciimmunol.abl4340.
36. Lopez, L.; Sang, P.C.; Tian, Y.; Sang, Y. Dysregulated Interferon Response Underlying Severe COVID-19. *Viruses* **2020**, *12*, 1433.
37. Zanoni, I. Interfering with SARS-CoV-2: Are interferons friends or foes in COVID-19? *Curr. Opin. Virol.* **2021**, *50*, 119–127.
38. Horvath, C.M. The Jak-STAT pathway stimulated by interferon alpha or interferon beta. *Sci. STKE* **2004**, *2004*, 10.
39. Yang, L.; Xie, X.; Tu, Z.; Fu, J.; Xu, D.; Zhou, Y. The signal pathways and treatment of cytokine storm in COVID-19. *Signal Transduction and Targeted Ther.* **2021**, *6*, 255.
40. Miorin, L.; Kehrer, T.; Sanchez-Aparicio, M.T.; Zhang, K.; Cohen, P.; Patel, R.S.; Cupic, A.; Makio, T.; Mei, M.; Moreno, E.; et al. SARS-CoV-2 Orf6 hijacks Nup98 to block STAT nuclear import and antagonize interferon signaling. *Proc. Natl. Acad. Sci.* **2020**, *117*, 28344–28354.
41. Xia, H.; Cao, Z.; Xie, X.; Zhang, X.; Chen, J.Y.; Wang, H.; Menachery, V.D.; Rajsbaum, R.; Shi, P.Y. Evasion of Type I Interferon by SARS-CoV-2. *Cell Rep.* **2020**, *33*, 108234.
42. Yuen, C.K.; Lam, J.Y.; Wong, W.M.; Mak, L.F.; Wang, X.; Chu, H.; Cai, J.P.; Jin, D.Y.; To, K.K.; Chan, J.F.; et al. SARS-CoV-2 nsp13, nsp14, nsp15 and orf6 function as potent interferon antagonists. *Emerg. Microbes. Infect.* **2020**, *9*, 1418–1428.
43. Chen, D.Y.; Khan, N.; Close, B.J.; Goel, R.K.; Blum, B.; Tavares, A.H.; Kenney, D.; Conway, H.L.; Ewoldt, J.K.; Chitalia, V.C.; et al. SARS-CoV-2 Disrupts Proximal Elements in the JAK-STAT Pathway. *J. Virol.* **2021**, *95*, e0086221.
44. Chen, W. A potential treatment of COVID-19 with TGF-β blockade. *Int. J. Biol. Sci.* **2020**, *16*, 1954–1955.
45. Stukalov, A.; Girault, V.; Grass, V.; Karayel, O.; Bergant, V.; Urban, C.; Haas, D.A.; Huang, Y.; Oubraham, L.; Wang, A.; et al. Multilevel proteomics reveals host perturbations by SARS-CoV-2 and SARS-CoV. *Nature* **2021**, *594*, 246–252.

46. Luo, W.; Li, Y.X.; Jiang, L.J.; Chen, Q.; Wang, T.; Ye, D.W. Targeting JAK-STAT Signaling to Control Cytokine Release Syndrome in COVID-19. *Trends Pharmacol. Sci.* **2020**, *41*, 531–543.
47. Aydemir, M.N.; Aydemir, H.B.; Korkmaz, E.M.; Budak, M.; Cekin, N.; Pinarbasi, E. Computationally predicted SARS-COV-2 encoded microRNAs target NFKB, JAK/STAT and TGFB signaling pathways. *Gene Rep.* **2021**, *22*, 101012.
48. Xiong, Y.; Liu, Y.; Cao, L.; Wang, D.; Guo, M.; Jiang, A.; Guo, D.; Hu, W.; Yang, J.; Tang, Z.; et al. Transcriptomic characteristics of bronchoalveolar lavage fluid and peripheral blood mononuclear cells in COVID-19 patients. *Emerg. Microbes. Infect.* **2020**, *9*, 761–770.
49. Fakhri, S.; Nouri, Z.; Moradi, S.Z.; Akkol, E.K.; Piri, S.; Sobarzo-Sánchez, E.; Farzaei, M.H.; Echeverría, J. Targeting Multiple Signal Transduction Pathways of SARS-CoV-2: Approaches to COVID-19 Therapeutic Candidates. *Molecules* **2021**, *26*, 2917.
50. Vallée, A.; Lecarpentier, Y.; Vallée, J.N. Interplay of Opposing Effects of the WNT/β-Catenin Pathway and PPARγ and Implications for SARS-CoV2 Treatment. *Front. Immunol.* **2021**, *12*, 666693.
51. Jin, Y.; Ji, W.; Yang, H.; Chen, S.; Zhang, W.; Duan, G. Endothelial activation and dysfunction in COVID-19: From basic mechanisms to potential therapeutic approaches. *Signal Transduct Target Ther.* **2020**, *5*, 293.
52. Chi, Y.; Ge, Y.; Wu, B.; Zhang, W.; Wu, T.; Wen, T.; Liu, J.; Guo, X.; Huang, C.; Jiao, Y.; et al. Serum Cytokine and Chemokine Profile in Relation to the Severity of Coronavirus Disease 2019 in China. *J. Infect. Dis.* **2020**, *222*, 746–754.
53. Kong, Y.; Han, J.; Wu, X.; Zeng, H.; Liu, J.; Zhang, H. VEGF-D: A novel biomarker for detection of COVID-19 progression. *Critical Care* **2020**, *24*, 373.
54. Hemmat, N.; Asadzadeh, Z.; Ahangar, N.K.; Alemohammad, H.; Najafzadeh, B.; Derakhshani, A.; Baghbanzadeh, A.; Baghi, H.B.; Javadrashid, D.; Najafi, S.; et al. The roles of signaling pathways in SARS-CoV-2 infection; lessons learned from SARS-CoV and MERS-CoV. *Arch. Virol.* **2021**, *166*, 675–696.
55. Vagapova, E.R.; Lebedev, T.D.; Prassolov, V.S. Viral fibrotic scoring and drug screen based on MAPK activity uncovers EGFR as a key regulator of COVID-19 fibrosis. *Sci. Rep.* **2021**, *11*, 11234.
56. Rex, D.A.B.; Dagamajalu, S.; Kandasamy, R.K.; Raju, R.; Prasad, T.S.K. SARS-CoV-2 signaling pathway map: A functional landscape of molecular mechanisms in COVID-19. *J. Cell. Commun. Signal* **2021**, 1–8. doi.: 10.1007/s12079-021-00632-4.
57. Suryawanshi, R.K.; Koganti, R.; Agelidis, A.; Patil, C.D.; Shukla, D. Dysregulation of Cell Signaling by SARS-CoV-2. *Trends Microbiol.* **2021**, *29*, 224–237.
58. Alfarouk, K.O.; Alhoufie, S.T.S.; Hifny, A.; Schwartz, L.; Alqahtani, A.S.; Ahmed, S.B.M.; Alqahtani, A.M.; Alqahtani, S.S.; Muddathir, A.K.; Ali, H.; et al. Of mitochondrion and COVID-19. *J. Enzyme Inhib. Med. Chem.* **2021**, *36*, 1258–1267.
59. Ganji, R.; Reddy, P.H. Impact of COVID-19 on Mitochondrial-Based Immunity in Aging and Age-Related Diseases. *Front. Aging Neurosci* **2020**, *12*, 614650.
60. Icard, P.; Lincet, H.; Wu, Z.; Coquerel, A.; Forgez, P.; Alifano, M.; Fournel, L. The key role of Warburg effect in SARS-CoV-2 replication and associated inflammatory response. *Biochimie* **2021**, *180*, 169–177.
61. Bordoni, V.; Tartaglia, E.; Sacchi, A.; Fimia, G.M.; Cimini, E.; Casetti, R.; Notari, S.; Grassi, G.; Marchioni, L.; Bibas, M.; et al. The unbalanced p53/SIRT1 axis may impact lymphocyte homeostasis in COVID-19 patients. *Int. J. Infect. Dis.* **2021**, *105*, 49–53.
62. Yuan, S.; Peng, L.; Park, J.J.; Hu, Y.; Devarkar, S.C.; Dong, M.B.; Shen, Q.; Wu, S.; Chen, S.; Lomakin, I.B.; et al. Nonstructural Protein 1 of SARS-CoV-2 Is a Potent Pathogenicity Factor Redirecting Host Protein Synthesis Machinery toward Viral RNA. *Mol. Cell* **2020**, *80*, 1055–1066.
63. Banerjee, A.K.; Blanco, M.R.; Bruce, E.A.; Honson, D.D.; Chen, L.M.; Chow, A.; Bhat, P.; Ollikainen, N.; Quinodoz, S.A.; Loney, C.; et al. SARS-CoV-2 Disrupts Splicing, Translation, and Protein Trafficking to Suppress Host Defenses. *Cell* **2020**, *183*, 1325–1339.
64. Lapointe, C.P.; Grosely, R.; Johnson, A.G.; Wang, J.; Fernández, I.S.; Puglisi, J.D. Dynamic competition between SARS-CoV-2 NSP1 and mRNA on the human ribosome inhibits translation initiation. *Proc. Natl. Acad. Sci. U. S. A.* **2021**, *118*. doi.: 10.1073/pnas.2017715118
65. Su, M.; Chen, Y.; Qi, S.; Shi, D.; Feng, L.; Sun, D. A Mini-Review on Cell Cycle Regulation of Coronavirus Infection. *Front. Vet. Sci.* **2020**, *7*, 586826.
66. Zhu, N.; Wang, W.; Liu, Z.; Liang, C.; Wang, W.; Ye, F.; Huang, B.; Zhao, L.; Wang, H.; Zhou, W.; et al. Morphogenesis and cytopathic effect of SARS-CoV-2 infection in human airway epithelial cells. *Nature Commun.* **2020**, *11*, 3910–3910.
67. Pei, R.; Feng, J.; Zhang, Y.; Sun, H.; Li, L.; Yang, X.; He, J.; Xiao, S.; Xiong, J.; Lin, Y.; et al. Host metabolism dysregulation and cell tropism identification in human airway and alveolar organoids upon SARS-CoV-2 infection. *Protein. Cell.* **2021**, *12*, 717–733.
68. Liu, Y.; Garron, T.M.; Chang, Q.; Su, Z.; Zhou, C.; Gong, E.C.; Zheng, J.; Yin, Y.; Ksiazek, T.; Brasel, T.; et al. Cell-type apoptosis in lung during SARS-CoV-2 infection. *bioRxiv* **2020,** in press.
69. Li, S.; Zhang, Y.; Guan, Z.; Li, H.; Ye, M.; Chen, X.; Shen, J.; Zhou, Y.; Shi, Z.L.; Zhou, P.; et al. SARS-CoV-2 triggers inflammatory responses and cell death through caspase-8 activation. *Signal Transduct Target Ther.* **2020**, *5*, 235.
70. Chan, J.F.; Zhang, A.J.; Yuan, S.; Poon, V.K.; Chan, C.C.; Lee, A.C.; Chan, W.M.; Fan, Z.; Tsoi, H.W.; Wen, L.; et al. Simulation of the Clinical and Pathological Manifestations of Coronavirus Disease 2019 (COVID-19) in a Golden Syrian Hamster Model: Implications for Disease Pathogenesis and Transmissibility. *Clin. Infect. Dis.* **2020**, *71*, 2428–2446.
71. Li, S.; Jiang, L.; Li, X.; Lin, F.; Wang, Y.; Li, B.; Jiang, T.; An, W.; Liu, S.; Liu, H.; et al. Clinical and pathological investigation of patients with severe COVID-19. *JCI Insight.* **2020**, *5*. doi.: 10.1172/jci.insight.138070

72. Paolini, A.; Borella, R.; De Biasi, S.; Neroni, A.; Mattioli, M.; Lo Tartaro, D.; Simonini, C.; Franceschini, L.; Cicco, G.; Piparo, A.M.; et al. Cell Death in Coronavirus Infections: Uncovering Its Role during COVID-19. *Cells* **2021**, *10*, 1585.
73. Raaben, M.; Posthuma, C.C.; Verheije, M.H.; te Lintelo, E.G.; Kikkert, M.; Drijfhout, J.W.; Snijder, E.J.; Rottier, P.J.; de Haan, C.A. The ubiquitin-proteasome system plays an important role during various stages of the coronavirus infection cycle. *J. Virol.* **2010**, *84*, 7869–7879.
74. Zhang, H.; Tu, J.; Cao, C.; Yang, T.; Gao, L. Proteasome activator PA28γ-dependent degradation of coronavirus disease (COVID-19) nucleocapsid protein. *Biochem. Biophys. Res. Commun.* **2020**, *529*, 251–256.

.
